# Loss of 15-lipoxygenase disrupts T_reg_ differentiation altering their pro-resolving functions

**DOI:** 10.1038/s41418-021-00807-x

**Published:** 2021-05-27

**Authors:** Raquel M. Marques, Maria Gonzalez-Nunez, Mary E. Walker, Esteban A. Gomez, Romain A. Colas, Trinidad Montero-Melendez, Mauro Perretti, Jesmond Dalli

**Affiliations:** 1 William Harvey Research Institute, Barts and The London School of Medicine and Dentistry, Queen Mary University of London, Charterhouse Square, London, UK; 2 Centre for Inflammation and Therapeutic Innovation, Queen Mary University of London, London, UK

**Keywords:** Enzymes, Chronic inflammation, T cells

## Abstract

Regulatory T-cells (T_regs_) are central in the maintenance of homeostasis and resolution of inflammation. However, the mechanisms that govern their differentiation and function are not completely understood. Herein, we demonstrate a central role for the lipid mediator biosynthetic enzyme 15-lipoxygenase (ALOX15) in regulating key aspects of T_reg_ biology. Pharmacological inhibition or genetic deletion of ALOX15 in T_regs_ decreased FOXP3 expression, altered T_reg_ transcriptional profile and shifted their metabolism. This was linked with an impaired ability of *Alox15*-deficient cells to exert their pro-resolving actions, including a decrease in their ability to upregulate macrophage efferocytosis and a downregulation of interferon gamma expression in Th1 cells. Incubation of T_regs_ with the ALOX15-derived specilized pro-resolving mediators (SPM)s Resolvin (Rv)D3 and RvD5_n-3 DPA_ rescued FOXP3 expression in cells where ALOX15 activity was inhibited. In vivo, deletion of *Alox15* led to increased vascular lipid load and expansion of Th1 cells in mice fed western diet, a phenomenon that was reversed when *Alox15-*deficient mice were reconstituted with wild type T_regs_. Taken together these findings demonstrate a central role of pro-resolving lipid mediators in governing the differentiation of naive T-cells to T_regs_.

## Introduction

Regulatory T-cells (T_regs_) are a subset of T-cells that play a central role in the maintenance of homeostasis. Defects in T_regs_ differentiation and/or function are linked with dysregulated lymphoproliferation, autoimmune diseases and chronic inflammation, including atherosclerosis [1–4]. This is because T_regs_ are tasked with preventing the unregulated activation of cells within both the innate and adaptive arms of the immune system [1–4]. Amongst the many biological actions exerted by these cells, T_regs_ play a central role in preventing the uncontrolled activation of effector CD4 and CD8 T-cells, limiting their ability to proliferate and the release of inflammatory cytokines, such as Interferon (IFN)-γ, Tumor Necrosis Factor (TNF)-α and Interleukin (IL)-17 [1, 5]. In addition, recent studies suggest that T_regs_ also exert pro-resolving actions by promoting the uptake of apoptotic cells by macrophages, a key step in the resolution of acute inflammation [4]. Notably, while the biology of T_regs_ has been studied extensively, the mechanisms that are involved in coordinating their differentiation from naive T-cells are not completely understood.

Recent studies highlight a role of lipid metabolizing enzymes, in particular enzymes involved the production and further metabolism of lipid mediators, in conferring T_regs_ their immunomodulatory activities [6, 7]. Amongst the main enzymes involved in the production of lipid mediators are lipoxygenases and cyclooxygenases [8]. Studies investigating the role of cyclooxygenases in T_reg_ biology found that cyclooxygenase (COX)-2, the inducible COX isoform, is upregulated during T_reg_ differentiation and inhibition of its activity reversed the immunosuppressive actions of these cells [6]. Recent studies also found that the prostaglandin (PG)E_2_ metabolizing enzyme, hydroxyprostaglandin dehydrogenase, is central for the immunosuppressive actions of visceral adipose tissue T_regs_ [7]. Curiously, little is known on the role and biological actions of lipoxygenases in T_regs_.

In humans there are four main ALOX isoforms that are denoted as ALOX5, ALOX12, ALOX15 and ALOX15b. These are non-heme iron-containing dioxygenases that catalyze the stereospecific oxygenation of essential fatty acids containing one or more 1,4-cis, cis pentadiene moieties [9]. These enzymes are involved in the production of both pro-inflammatory and protective mediators. For example, ALOX5 is the initiating enzyme in the biosynthesis of leukotrienes, including leukotriene (LT)B_4_, a potent phagocyte chemoattractant [8] that was recently suggested to negatively regulate the expression of the key T_reg_ transcription factor FOXP3 [10]. This enzyme is also involved in the production of the host protective Lipoxins (LX) that regulate phagocyte trafficking to the site of inflammation [11], downregulate inflammatory cytokine production by cells from both the innate and adaptive arms of the immune system [2, 12] and upregulate FOXP3 expression in T_regs_ [2]. Similarly, ALOX15 is involved in the biosynthesis of the pro-inflammatory Eoxins, which promote vascular leakage [13], and is the initiating enzyme in the protectin pathway [14, 15]. The latter mediators exert tissue protective and reparative actions by regulating innate responses, including those of macrophages [14, 16–18].

Of note, to date it is unknown whether ALOX enzymes are expressed in T_regs_ and whether these play a role in coordinating the differentiation of these cells from naive T-cells. Herein we demonstrate that human T_regs_ express ALOX5, ALOX12 and ALOX15. Using LC-MS/MS-based lipid mediator profiling, we found that these enzymes were functionally active producing both pro-resolving and pro-inflammatory mediators. Furthermore, inhibiting the activity or expression of ALOX15 using pharmacological and genetic approaches resulted in a marked shift in the T_reg_ metabolic profile as well as a loss in their regulatory and pro-resolving actions when assessed both in vitro and in vivo. These findings highlight a fundamental role for ALOX15 in coordinating T_regs_ differentiation and function.

## Results

### ALOX15 is upregulated during T_reg_ differentiation

**Fig. 1:**
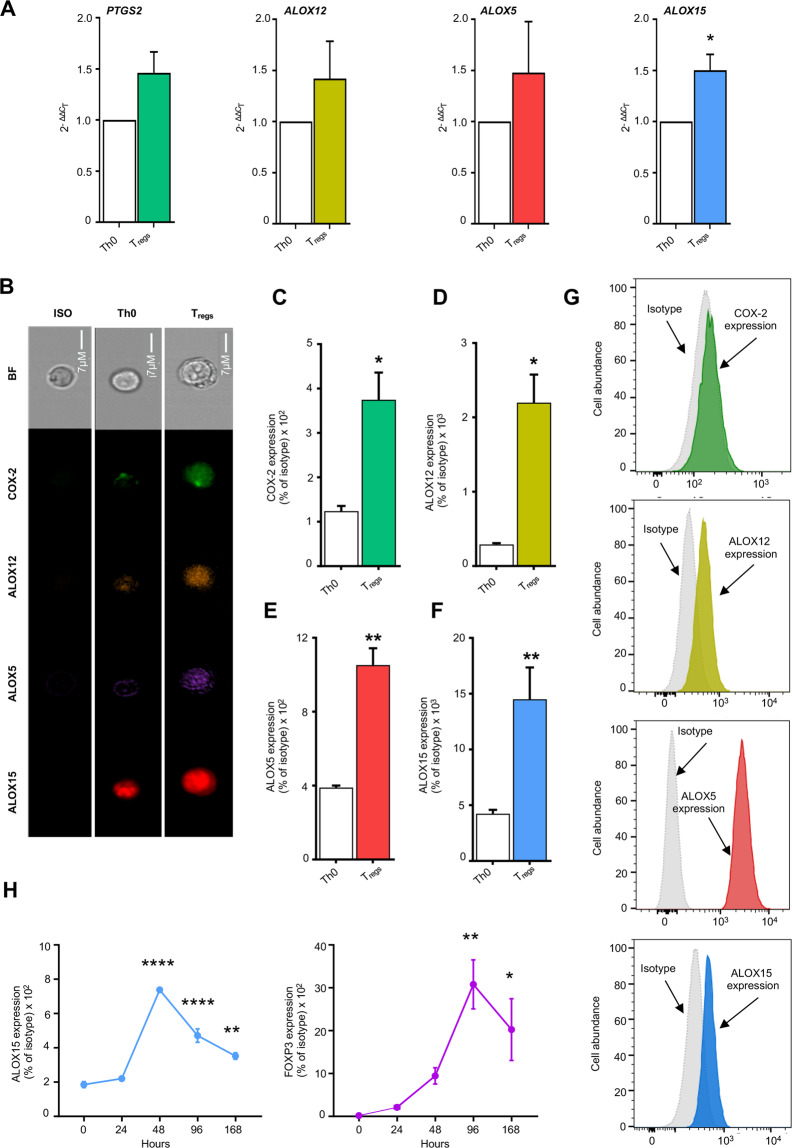
Upregulation of ALOX15 in human T_regs_. Naive CD4^+^ T lymphocytes (Th0) were isolated from healthy volunteers, differentiated to T_regs_ (see methods for details) and the expression of lipid mediator biosynthetic enzymes was determined (**A**) using quantitative real-time PCR (**B**–**F**) using fluorescently labeled antibodies and flow cytometry and compared to the expression observed in naïve CD4^+^ T-cells (Th0). Protein expression in the two cell types was then normalized against the signal obtain for the respective isotype. (**G)** Representative histogram plots depicting the expression of ALOX and COX2 enzymes in natural T_regs_ isolated from human peripheral blood. (**H)** Temporal regulation of ALOX15 and FOXP3 during T_reg_ differentiation was determined using fluorescently labeled antibodies and flow cytometry. Results are mean ± s.e.m. of *n* = 4–5 donors from two distinct experiments and expressed for **A** as fold change from Th0 (2^−ΔΔC^_T_) and for **B**–**H** as mean fluorescence intensity units (MFI); *p < 0.05, **p < 0.01, One sample *t* test for A or Unpaired *t* test with Welch’s correction for **C**–**F**; For **G** ***p < 0.001, 1-way ANOVA with Tukey multiple comparisons test.

To investigate whether T_regs_ expressed ALOX enzymes, we obtained T_regs_ from human CD4^+^ T-cells and assessed expression of ALOX5, ALOX12 and ALOX15 using both quantitative real-time PCR and flow cytometry, using COX-2 as positive control [6]. Here we found that ALOX5, ALOX12, ALOX15 and COX-2, were expressed in human naïve CD4^+^ T cells at both mRNA (Fig. 1A) and protein level (Fig. 1B–F). Once differentiated to T_regs_, both mRNA and protein expression of all four enzymes were increased (Fig. 1B–F). Similar observations were also made in murine T_regs_ where we observed an upregulation of ALOX5, and ALOX15 in T_regs_ when compared with naïve CD4^+ ^T-cells (Supplementary Fig. S2).

We next evaluated whether these enzymes were also expressed in natural T_regs_. Flow cytometric evaluation of circulating CD3^+^CD4^+^CD25^+^CD127^lo^FOXP3^+^ cells demonstrated that all three ALOX enzymes were robustly detected (Fig. 1G). Given the central role that ALOX15 plays in lipid mediator biosynthesis [15, 18, 19], we next assessed the time course for ALOX15 upregulation in relation to FOXP3 expression during the course of T_reg_ differentiation. Here we found that expression of ALOX15 reaches a maximum at the 48h time-point whereas FOXP3 reaches a maximum at 96h time-point (Fig. 1H).

**Fig. 2:**
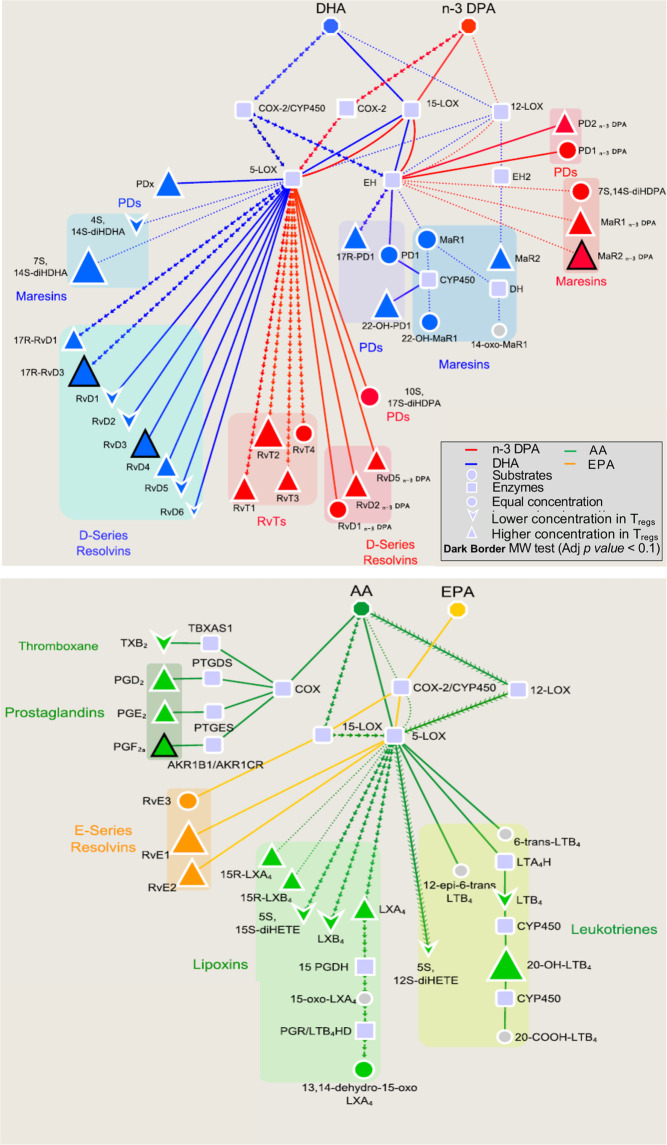
ALOX15-derived mediators are increased in T_regs_. T_regs_ and naïve CD4^+^ T lymphocytes (Th0) were obtained from healthy volunteers (*see methods for details*) and placed in ice-cold methanol containing deuterium labeled internal standards. Lipid mediators were then extracted, identified and profiled using LC-MS/MS–based lipid mediator profiling. Pathway analysis for the differential expression of mediators from the DHA and n-3 DPA **(top panel)**, and EPA and AA **(bottom panel)** bioactive metabolomes in T_regs_ when compared to naive CD4^+^ T-cells. Statistical differences between the normalized concentrations (expressed as the fold change) of the lipid mediators from the T_regs_ and Th0 cells were determined using Mann–Whitney test followed by a multiple comparison correction using Benjamini–Hochberg procedure. Results are representative of *n* = 4 donors from two distinct experiments.

Having found that T_regs_ express ALOX enzymes, we next questioned whether these cells produced lipid mediators and whether production of these molecules was modulated during the T_reg_ differentiation process. Lipid mediator profiling demonstrated that T_regs_ produced mediators from all four major bioactive metabolomes that include docosahexaenoic acid (DHA) and n-3 docosapentaenoic acid (n-3 DPA)-derived resolvins and protectins as well as arachidonic acid (AA)-derived lipoxins, leukotrienes and prostaglandins. Furthermore, the concentrations of ALOX15-derived mediators were upregulated when compared with lipid mediator concentrations found in naïve CD4^+^ T-cells (Fig. 2, Supplementary Table S1).

### Inhibition of ALOX15 during the early stages of T_reg_ differentiation reduces FOXP3 expression and alters T_reg_ phenotype

**Fig. 3:**
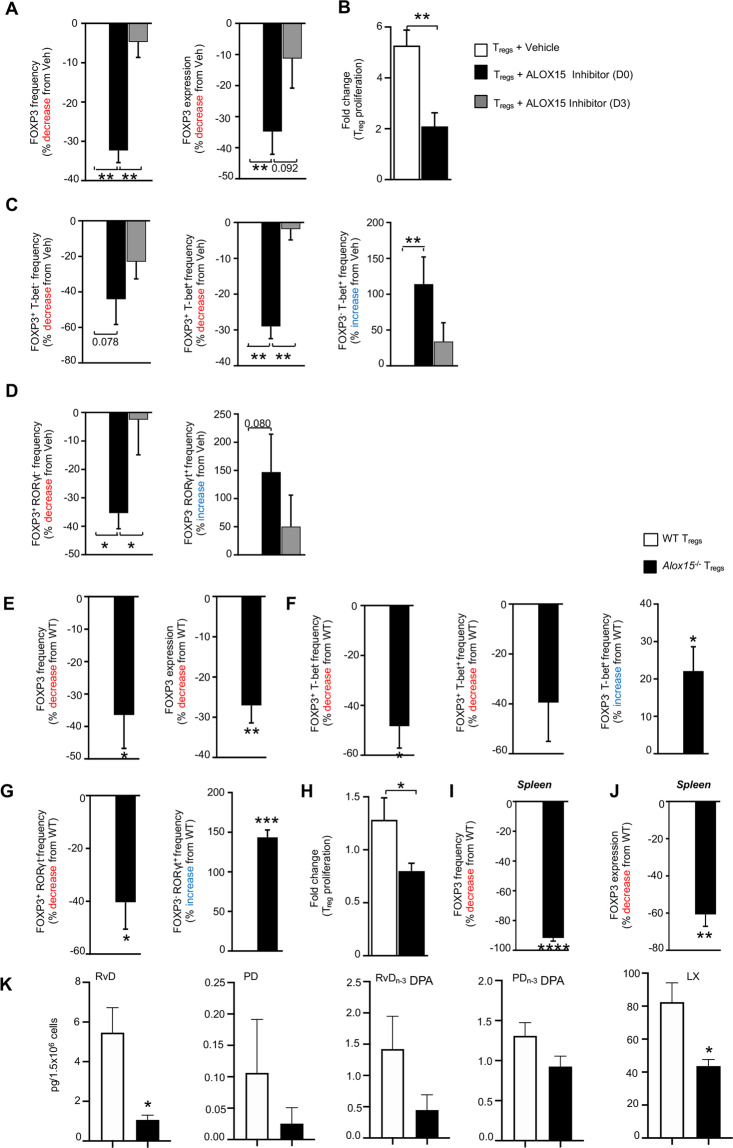
ALOX15 inhibition decreases FOXP3 expression in T_regs_ and alters their phenotype. T_regs_ were obtained from healthy volunteers and incubated with an ALOX15 inhibitor (PD146176; 5 μM) on day 0 (D0) or day 3 (D3) or vehicle (0.01% DMSO). (**A)** The number of FOXP3 positive cells (*left panel*) and the expression of FOXP3 (*right panel*), (**B)** Cellular proliferation, together with the number of (**C**) FOXP3 and/or T-bet expressing cells and (**D**) FOXP3 and/or RORγt expressing cells was determined using flow cytometry and fluorescently labeled antibodies. Results are expressed mean ± s.e.m of % change from vehicle control. *n* = 10 healthy volunteers from two distinct experiments. (**E–H)** Mouse T_regs_ were obtained from WT and *Alox15*^*−/−*^ mice. (**E)** The number of FOXP3 positive cells (*left panel*) and the expression of FOXP3 (*right panel*), together with the number of (**F**) FOXP3 and/or T-bet expressing cells and (**G**) FOXP3 and/or RORγt expressing cells and (**H**) Cellular proliferation was determined using flow cytometry and fluorescently labeled antibodies. Splenocytes were isolated from WT and *Alox15*^*−/−*^ mice and (**I**) the number of FOXP3 positive cells and (**J**) the expression of FOXP3 was determined using flow cytometry. *n* = 4 mice per group from two distinct experiments. For **A**–**D** *p < 0.05, **p < 0.01, using One sample *t* test and Unpaired *t* test with Welch’s correction. For **E**–**J** *p < 0.05, **p < 0.01, ***p < 0.001, ****p < 0.0001; One sample *t* test. (**K)** Mouse T_regs_ were obtained from WT and *Alox15*^*−/−*^ mice and the concentrations of ALOX15-derived lipid mediators was determined using LC-MS/MS-based lipid mediator profiles. Results are mean ± sem. *n* = 3–4 mice per group **A**–**D** *p < 0.05 Unpaired *t* test with Welch’s correction.

Having established that ALOX15-derived lipid mediators were upregulated in T_regs_, we next questioned whether these mediators were involved in the T_reg_ differentiation program. For this purpose, we incubated naïve CD4^+^ T-cells with an ALOX15 inhibitor and assessed the expression of FOXP3, the characteristic T_reg_ transcription factor, in the resultant cells. Inhibition of ALOX15 throughout the time course of T_reg_ differentiation led a decrease in the production of ALOX15-derived SPM including the docosahexaenoic acid (DHA)-derived Resolvin D3 (RvD3) and n-3 docosapentaenoic acid-derived resolvin D5 (RvD5_n-3 DPA_; Supplementary Table S2). This decrease in ALOX15-derived mediators was linked with a reduction in the expression of FOXP3 (Fig. 3A and Supplementary Fig. S1A, B) as well as T_reg_ proliferation (Fig. 3B). Given that ALOX15 expression was upregulated early (Day 2) in the course of T_reg_ differentiation, we next questioned whether inhibiting the activity of the enzyme at a later stage during the T_reg_ differentiation also reduced FOXP3 expression. Of note, inhibition of ALOX15 from day 3 through to day 7, the end of the differentiation time course, did not significantly alter FOXP3 expression (Fig. 3A, C, D). These results suggest that ALOX15-derived lipid mediator production early on in the differentiation process was central in governing T_reg_ differentiation.

In order to further characterize the phenotype of T_regs_ following ALOX15 inhibition, we also assessed expression of transcription factors linked with T-effector function namely T-bet and Rorγt, observing that both were upregulated in these culture conditions (Fig. 3C, D and Supplementary Fig. S1A, B). This observation was further supported when we quantified expression of these transcription factors in T_regs_ obtained by differentiating naïve CD4^+^ T-cells from *Alox15*-deficient mice. Here FOXP3 expression was reduced (Fig. 3E), whereas expression of Rorγt and T-bet was upregulated in *Alox15*^*−/−*^ cells (Fig. 3F, G). These changes in transcription factor expression were linked with a reduction in cell proliferation in *Alox15*^*−/−*^ cells when compared with their WT counterparts (Fig. 3H). Of note, these results were also corroborated in vivo, where in spleens from *Alox15*^*−/−*^ *mice*, we observed a significant reduction in both the expression of FOXP3 in T_regs_ as well as in the frequency of splenic T_regs_ when compared with WT mice (Fig. 3I, J). Notably, this reduction in FOXP3 expression in *Alox15*^−/−^ T_regs_ was also linked with a reduction in ALOX15-derived SPM concentrations (Fig. 3K and Supplementary Table S3).

**Fig. 4:**
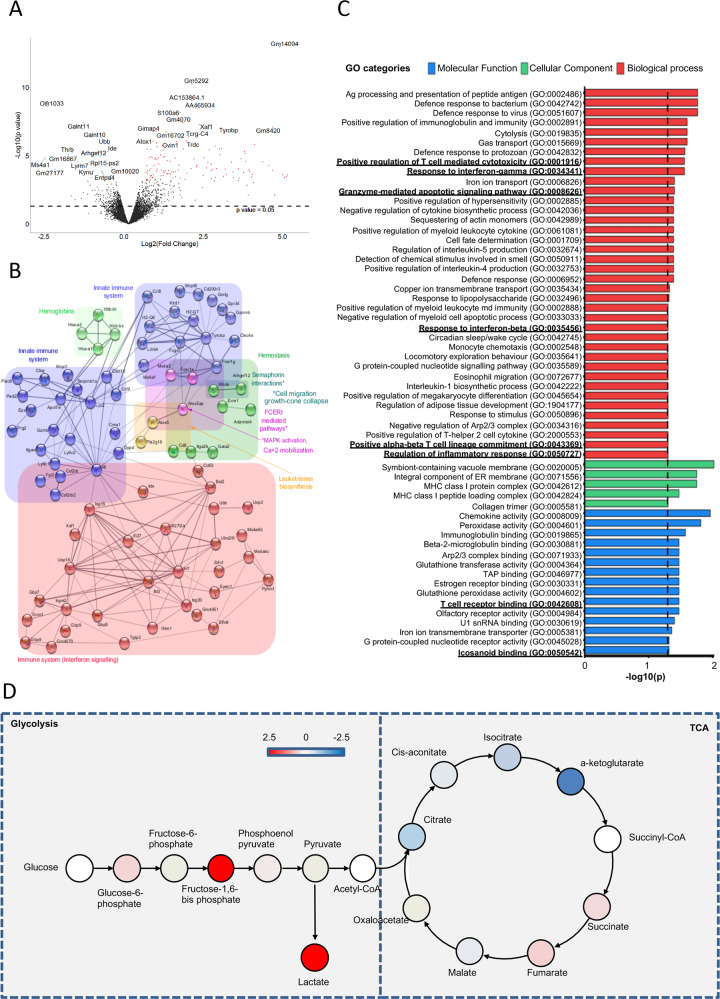
*Alox15*^*−/−*^ T_regs_ display altered transcriptional and metabolic profiles. WT and *Alox15*^*−/−*^ CD4^+^ naïve T-cells were differentiated to T_regs_ (see methods for details) and their transcriptional profile was determined 2 days after the initiation of differentiation using MACEseq. (**A)** Volcano plot highlighting the significantly upregulated (red) and downregulated (blue) genes in T_regs_ from *Alox15*^*−/−*^ mice. (**B)** Enriched pathways identified in differentially expressed genes for *Alox15*^*−/−*^ T_regs_ using Reactome and KEGG pathways. Pathways were illustrated using the STRING database. (**C)** Gene Ontology (GO) term enrichment analysis for the GO categories biological process, cellular component and molecular function of differentially expressed genes in T_regs_ from *Alox15*^*−/−*^ mice. Significance was determined after Benjamini–Hochberg correction (adjusted *p* value of <0.05). Results are representative of *n* = 4 *Alox15*^*−/−*^ mice. (**D)** WT and *Alox15*^*−/−*^ CD4^+^ naïve were differentiated to T_regs_ (see methods for details). On day 7 the concentrations of metabolites from the glycolysis and TCA pathways was determined using liquid chromatography-tandem mass spectrometry. Results are expressed as the fold change of metabolites identified in *Alox15*^*−/−*^ T_regs_ versus WT T_regs_. Red illustrates metabolites that were upregulated in *Alox15*^*−/−*^ T_regs_ and blue metabolites that were downregulated in these cells. For metabolites denoted in white circles no differences were found between the two groups whilst those denoted in gray were not measured. Results are representative of *n* = 4 mice per group from two distinct experiments.

To establish if these changes were limited to the expression of transcription factors or were linked with a wider alteration of cellular phenotype, we next conducted transcriptomic analysis of T_regs_ obtained from WT and *Alox15*^−/−^ mice. Differential gene expression analysis demonstrated a significant alteration in genes associated with several cellular functions that included cellular metabolism and immunity. This included an upregulation of genes involved in glycolysis and interferon signaling pathway including *Interferon regulatory factor 7, Interferon Induced Protein With Tetratricopeptide Repeats 3* and *Interferon Induced Transmembrane Protein 1* (Supplementary Table S4). These changes were coupled with a downregulation of genes involved in TCA cycle and MAP kinase signaling pathways including *Ubiquitin B* (Fig. 4A–C, Supplementary Table S4).

Having observed a differential regulation in metabolic genes in cells from *Alox15*^−/−^ mice and given the central role that these pathways play in T_reg_ function, we next assessed whether cellular metabolism was also altered in *Alox15*^−/−^ T_regs_. For this purpose, we measured steady state concentration of metabolites within both the glycolytic pathway and the TCA cycle using liquid chromatography tandem-mass spectrometry. Here we found an upregulation of metabolites from the glycolytic pathway that was linked with an overall downregulation in the concentrations of metabolites from the TCA cycle in *Alox15*^*−/−*^ when compared with WT T_regs_ (Fig. 4D). Upregulation of glycolytic metabolites was also observed following pharmacological inhibition of ALOX15 in human T_regs_ (Supplementary Fig. S3A). These findings demonstrate that inhibition of ALOX15 during T_reg_ differentiation leads to a disruption in both the transcriptional program and specific metabolic pathways in these cells.

### Inhibition of ALOX15 in T_regs_ impairs their regulatory functions

**Fig. 5:**
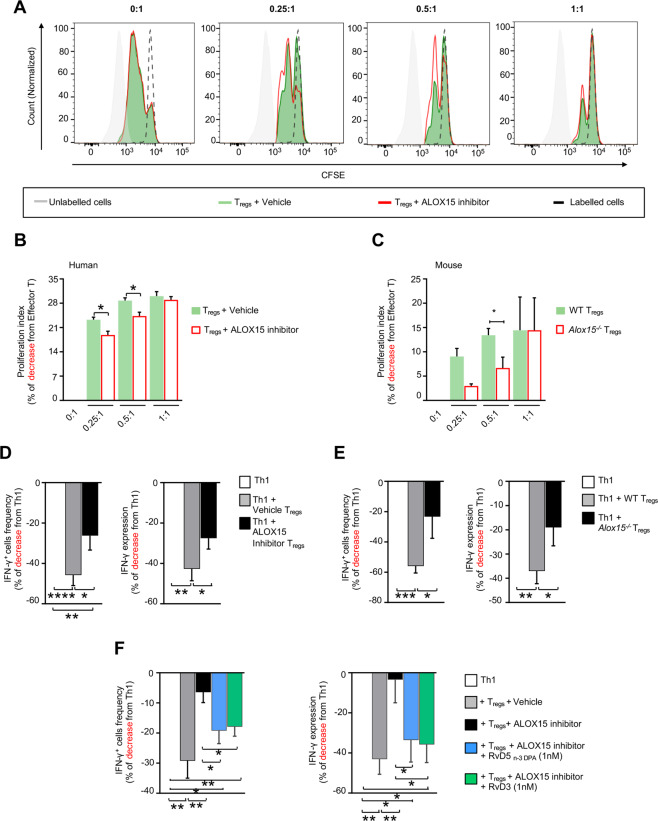
Inhibition of ALOX15 activity in T_regs_ limits their regulatory functions. T_regs_ were differentiated from (**A** and **B**) healthy volunteers CD4^+^ T-cells or (**C**) mouse CD4^+^ T-cells (see methods for details) and incubated with CFSE-labeled CD4^+^CD25^−^ (T_effectors_) at the indicated ratios. After 3 days proliferation was determined using flow cytometry. (**A)** Representative histogram plots used to determine proliferation, (**B)** human T_effector_ proliferation, (**C)** mouse T_effector_ proliferation. Results are mean ± s.e.m. of % change from Effector T cells; *n* = 3 per group; *p < 0.05, Unpaired *t* test with Welch’s correction. (**D–F)** Th1 cells were differentiated from CD4^+^ T lymphocytes (see methods for details) and incubated without or with T_regs_ (1:0,5 ratio) for 18 h, after which GolgiSTOP was added and IFN-γ expression determined 6 h later using flow cytometry. Reduction in the frequency of IFN-γ expressing cells (left panel) and in the expression of IFN-γ per cell (right panel) for (**D**) human and (**E**) mouse Th1 cells. (**F)** Human T_regs_ were obtained as detailed above, in the presence or absence of ALOX15 inhibitor (5 µM) and with RvD3, RvD5_n-3 DPA_ (1 nM) each or vehicle (0.1% EtOH; 37 °C; 7 days). Cells were then incubated with Th1 cells for 24 h. IFN-γ expression was determined using flow cytometry. Results display the reduction in the frequency of IFN-γ expressing cells (left panel) and in the expression of IFN-γ per cell (right panel). Results are mean ± s.e.m and expressed as percent change from Th1 cells incubated alone. *n* = 8 donors for human cells and 6 mice for mouse cells from at least 2 distinct experiments. For **D**–**F** *p < 0.05, **p < 0.01, ***p < 0.001, ****p < 0.0001, One sample *t* test or Unpaired *t* test with Welch’s correction.

Having observed a shift in T_reg_ phenotype toward an effector phenotype when ALOX15 activity or expression was inhibited, we next investigated whether such a modulation was functionally relevant. To this end, we first evaluated whether these cells were able to regulate effector T-cell proliferation, a key biological action exerted by T_regs_. T_regs_ differentiated using conventional conditions inhibited T-effector cell proliferation as determined using flow cytometry, whereas cells differentiated in the presence of an ALOX15 inhibitor displayed a significantly reduced ability to inhibit T-cell proliferation (Fig. 5A, B). We also found that cells differentiated in the presence of an ALOX15 inhibitor displayed a significantly reduced ability to inhibit the expression of IFNγ in Th1 cells but not of IL-17A in Th17 cells (Fig. 5D, and Supplementary Figs. S1,C-F and S3B). These results were also replicated with *Alox15*^*−/−*^ cells that displayed a reduced ability to regulate T-effector cell proliferation (Fig. 5C) and IFNγ expression but not of IL-17A in Th17 cells (Fig. 5E and Supplementary Fig. S3C).

In order to further evaluate the role of ALOX15-derived lipid mediators in regulating T_reg_ function, we next appraised which mediators were increased during the early stages of T_reg_ differentiation. Here we found that several ALOX15-derived mediators from the DHA and n-3 DPA bioactive metabolomes, including RvD5_n-3 DPA_ and RvD3 were upregulated at the 24h time-point (Supplementary Fig. S4, Supplementary Table S5). Moreover, incubation of naive CD4^+^ T-cells with either one of these lipid mediators rescued the ability of T_regs_ differentiated in the presence of an ALOX15 inhibitor to regulate IFNγ expression in Th1 cells (Fig. 5F). Thus, these findings support a role for the ALOX15-derived lipid mediators RvD5_n-3 DPA_ and RvD3 in T_reg_ differentiation.

**Fig. 6:**
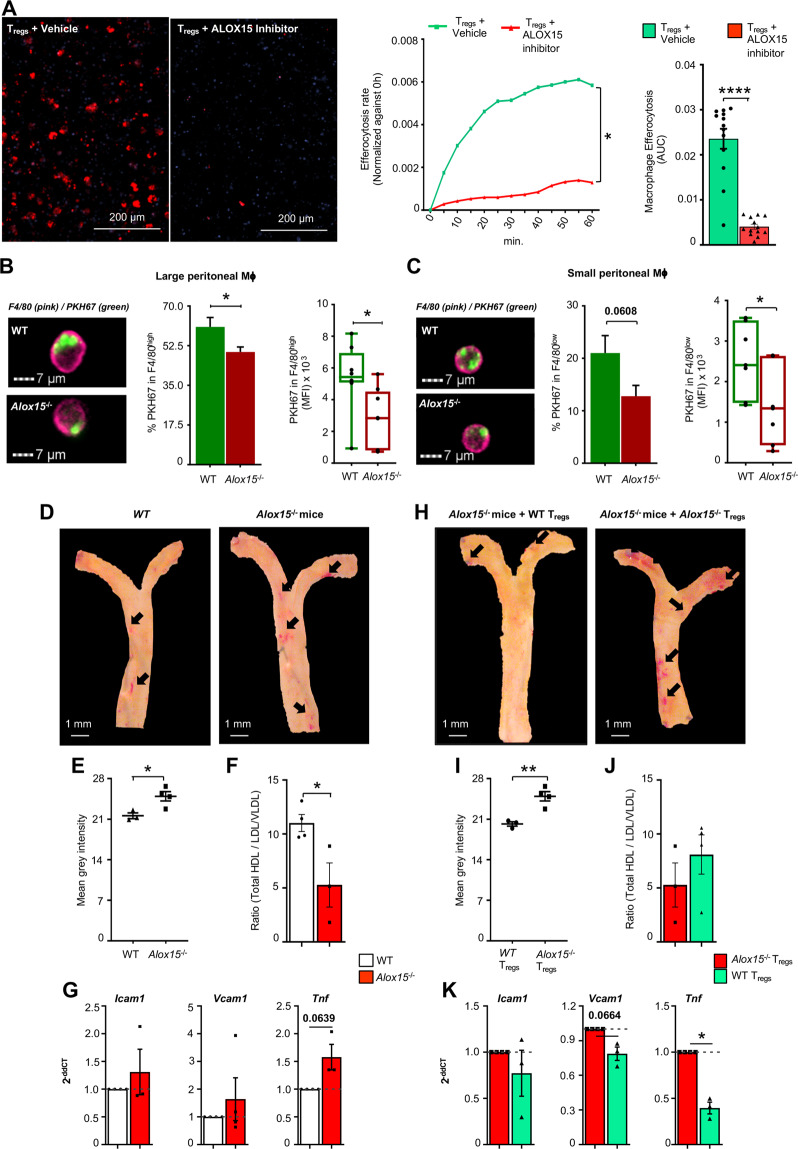
ALOX15^−/−^ T_regs_ display dysregulated pro-resolving actions in vivo. **(A)** Human monocyte-derived macrophages were obtained from peripheral blood monocytes (see methods for details). These were incubated with T_regs_ differentiated in the presence or absence of an ALOX15 inhibitor (5 µM) for 4 days at a ratio of 4:1 respectively. Macrophages were then harvested, incubated with fluorescently labeled apoptotic cells (1:3 ratio) and efferocytosis was assessed in real-time using High content Imaging. (*Left panels*) representative immunofluorescence staining of labeled apoptotic cells (red) and nuclei (blue) taken at 60 min, (*middle panel*) kinetics of apoptotic cell uptake, (*right panel*) quantitation of apoptotic cell uptake measuring the area under the curve (AUC). Results are mean ± s.e.m. and expressed as change in signal intensity recorded at baseline (0 min); *n* = 5 healthy volunteers; *p < 0.05, 2-way ANOVA with Tukey multiple comparisons test; ****p < 0.0001, using Mann–Whitney test. (**B**, **C)** WT and *Alox15*^*−/−*^ naive CD4^+^ T-cells were differentiated to T_regs_ (see methods for details). Peritonitis was initiated in RAG^−/−^ mice with 0.1 mg of zymosan administered via i.p injection. After 30 h, 3 × 10^5^ WT or *Alox15*^*−/−*^ T_regs_ were injected i.p. Four days later 6 × 10^6^ PKH67-labeled apoptotic cells were injected i.p., after 1-h peritoneal macrophages were collected and efferocytosis assessed using flow cytometry. (*left panel*) representative images (*central panel*) abundance of macrophage positive for apoptotic cells (*right panel*) amount of apoptotic cells taken up per cell for (**B**) large peritoneal macrophages (**C**) small peritoneal macrophages. Results are mean ± s.e.m.; *n* = 8 mice per group. *p < 0.05, Unpaired *t* test with Welch’s correction. (**D–K)** WT and *Alox15*^*−/−*^ mice were fed Western Diet for 8 weeks. Aortic arches were then harvested for real-time PCR quantification of inflammatory markers, and lesions were stained using Oil red O and quantified using ImageJ. Blood was also collected for cholesterol quantification in the plasma. (**D)** Representative images for aortic arches obtained from WT and *Alox15*^*−/−*^ mice (**E**) Aortic lesion quantitation. (**F)** Ratio of Total HDL versus LDL/VLDL. (**G)** *Icam1*, *Vcam1* and *Tnf* gene expression. (**H–K)** WT and *Alox15*^*−/−*^ T_regs_ were differentiated as outlined above and then transferred to *Alox15*^*−/−*^ mice (3 × 10^5^ cells per mouse) via i.v. injection. Mice were then fed Western Diet for 8 weeks and aortic arches and blood were collected for further analysis as in **E**–**G**. (**H)** Representative images for aortic arches obtained from *Alox15*^*−/−*^ mice administered WT and *Alox15*^*−/−*^ T_regs_ **(I)** Aortic lesion quantitation. (**J)** Ratio of Total HDL versus LDL/VLDL. (**K)** *Icam1*, *Vcam1* and *Tnf* gene expression. Results are means ± s.e.m.; *n* = 4 mice per group from two distinct experiments and expressed for **E**, **I** as mean gray intensity; **F**, **J** as the ratio between HDL and LDL/VLDL concentration; **G**, **K** as fold change from WT or Alox15^−/−^ mice that received Alox15^−/−^ T_regs_ (2^−ΔΔC^_T_); *p < 0.05, **p < 0.01, Unpaired *t* test with Welch’s correction for **E**, **F** and **I**, **J**; One sample *t* test for **G** and **K**.

Recent studies demonstrate that T_regs_ promote macrophage efferocytosis, a key biological action in the resolution of inflammation [4, 14]. Thus, we next investigated whether such a property was affected when ALOX15 activity was inhibited during T_reg_ differentiation. Here we found that T_regs_ treated with an ALOX15 inhibitor were less efficient at upregulating macrophage efferocytosis when compared to T_regs_ incubated with vehicle alone (Fig. 6A). To test if this impaired ability of T_regs_ derived from *Alox15*-deficient T-cells was also translated in vivo, we administered WT and *Alox15*-deficient T_regs_ to RAG^−/−^ mice, that are naturally deficient in T-cells, and assessed their ability to regulate macrophage efferocytosis of fluorescently labeled apoptotic cells. Macrophages from mice given *Alox15*-deficient T_regs_ displayed a reduced ability to uptake apoptotic cells when compared with macrophages from mice given WT T_regs_ (Fig. 6B, C). This was linked with a differential expression of several macrophage phenotypic markers, including MHC-II and CD11b in macrophages collected from mice reconstituted with *Alox15*^*−/−*^ T_regs_ (Supplementary Fig. S5).

### *Alox15*^*−/−*^ mice display increased vascular inflammation that is rescued by WT T_regs_

Vascular inflammation is associated with enhanced Th1 activity, altered T_reg_ function and impaired macrophage efferocytosis contributing to lipid accumulation in the vascular wall [4, 20]. Therefore, we next questioned whether mice deficient in *Alox15* displayed increased markers of vascular inflammation when fed an atherogenic diet in comparison to WT mice. Assessment of Oil red O staining, a marker of lipid load in the vascular wall [21, 22] following 8 weeks of western diet demonstrated enhanced vascular lipid load in *Alox15*-deficient mice when compared to WT mice (Fig. 6D, E). *Alox15*-deficient mice also presented with a significantly lower ratio of HDL to LDL/VLDL ratio in the plasma, and an increased expression of *Tnf-α* in the aortic tissues (Fig. 6F, G). These changes in systemic and tissue markers were linked with an increased activation of effector T-cells in peripheral blood cells as demonstrated by an increase in the expression of CD69 and IFNγ as well as the transcription factor T-bet (Supplementary Fig. S6A). This increase was also observed in splenic T-cells from *Alox15*^*−/−*^ mice, where we found an upregulation of IFNγ in splenic T-cells (Supplementary Fig. S6B).

To determine the contribution of T_regs_ to the observed increased pathology in *Alox15*^−/−^ mice, we adoptively transferred WT or *Alox15*^*−/−*^ T_regs_ and assessed disease activity in these mice after 8 weeks of western diet. Here we found that transfer of WT T_regs_ led to a significant downregulation of Oil red O staining in the aortic arch of *Alox15*^−/−^ mice when compared with mice that received *Alox15*^−/−^ T_regs_ (Fig. 6H, I). We also found an increased HDL to LDL/VLDL ratio in the plasma, and a decrease in *Icam-1*, *Vcam-1* and *TNF-α*, with changes reaching statistical significance for *Tnf-α* (Fig. 6J, K) in mice that received WT T_regs_. Furthermore, the transfer of WT T_regs_ was linked with a reduction in the number of activated and effector T-cells as measured by i) a reduction in the number of CD127 positive cells, ii) a reduction in IFNγ expression in circulating CD4^+^ T-cells as well as iii) a reduction in the expression of the Th1 transcription factor T-bet in both circulating and splenic CD4^+^ T-cells (Supplementary Fig. S6C, D).

### FOXP3 regulates ALOX15 expression during T_reg_ differentiation

**Fig. 7:**
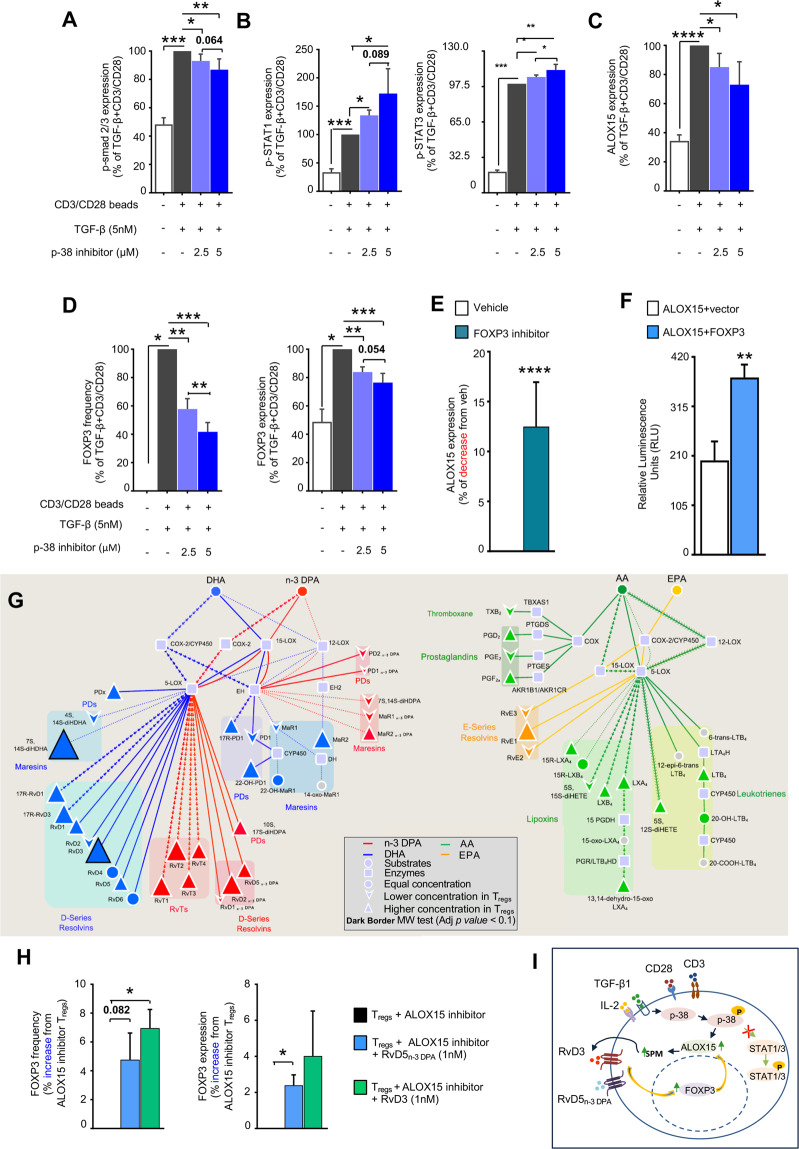
Increased ALOX15-derived lipid mediators by T_reg_ precursors leads to FOXP3 upregulation. **(A–D)** CD4^+^ T-cells were isolated from healthy volunteers and incubated for 30 min with p38 inhibitor (SB202190) or vehicle (0.01% DMSO), then with TGF-β+CD3/CD28 for (**A**, **B**) 12 h for the expression of (**A**) p-smad2/3, (**B**) p-STAT1 and p-STAT3, (**C**, **D**) or for 24 h and the expression of (**C**) ALOX15 and (**D**) FOXP3 assessed using flow cytometry. Results are mean ± s.e.m. and expressed as percent change from cells incubated with TGF-β+CD3/CD28 alone; *n* = 4–6 healthy volunteers; *p < 0.05, **p < 0.01, ***p < 0.001, ****p < 0.0001, One sample *t* test or Unpaired *t* test with Welch’s correction. (**E)** CD4^+^ T-cells were incubated with a FOXP3 inhibitor peptide (P60 peptide; 100 µM) or vehicle (0.01% DMSO) and cells differentiated to T_regs_ (see methods for details) for 7 days. ALOX15 expression was determined using flow cytometry. (**F)** HEK 293 cells were co- transfected with a vector containing the promoter region of *ALOX15* tagged with luciferase and either a vector containing a *FOXP3* or a control vector and luminescence was determined after 24 h. Results are mean ± s.e.m. *n* = 7 cell preparations from three independent experiments. For **E** ***p < 0.0001, One sample *t* test, For **F** **p < 0.01, Unpaired *t* test with Welch’s correction. (**G)** CD4^+^ T-cells were isolated from healthy volunteers and incubated with or without the T_reg_ differentiation cocktail for 1 h (37 °C). Incubations were quenched and lipid mediator profiles determined using lipid mediator profiling. Pathway analysis for the differential expression of mediators from the (*left panel*) DHA and n-3 DPA, and (*right panel*) EPA and AA bioactive metabolomes in cells incubated with the T_reg_ differentiation cocktail when compared to Th0 cells incubated in media alone. Statistical differences between the normalized concentrations (expressed as the fold change) of the lipid mediators from the cells incubated with T_reg_ cocktail and Th0 cells were determined using a Mann–Whitney test followed by a multiple comparison correction using Benjamini–Hochberg procedure. Results are representative of *n* = 6 donors from three distinct experiments. (**H)** Human T_regs_ were obtained as detailed above, in the presence or absence of ALOX15 inhibitor (5 µM) and with RvD3, RvD5_n-3 DPA_ (1nM) each or vehicle (0.1% EtOH; 37 °C; 7 days). Abundance of FOXP3 positive cells (*left panel*) and expression of FOXP3 per cell (*right panel*). Results are mean ± s.e.m. and expressed as percent change from T_regs_ + ALOX15 inhibitor, *n* = 4 donors from two independent experiments. *p < 0.05, One sample *t* test or Unpaired *t* test with Welch’s correction. (**I)** Scheme summarizing the proposed ALOX15-initiated T_reg_ differentiation pathway.

Since inhibition of ALOX15 activity or deletion of the enzyme leads to T_regs_ that displayed impaired regulatory actions, we next investigated the mechanisms linking FOXP3 and ALOX15. Having observed that inhibition of ALOX15 activity at Day 3 did not influence T_reg_ phenotype, we focused on the first 24 h of the T_reg_ differentiation time course. Given that TGF-β is known to regulate FOXP3 expression [23, 24], we assessed whether disruption of the TGF-β signaling pathway interfered with ALOX15 expression. Indeed, inhibition of p38 reduced the expression of pSMAD2/3, transcription factors known to regulate the expression of FOXP3 [25–27], upregulated p-STAT1 and p-STAT3 (Fig. 7A, B), and downregulated the expression of both ALOX15 and FOXP3 (Fig. 7C, D). Notably, inhibition of ERK signaling which can mediate the biological actions of TGF-β, did not reduce FOXP3 or ALOX15 expression (Supplementary Fig. S7A, B). Thus, these findings implicate SMAD2/3 phosphorylation as downstream signaling pathways in regulating the expression of FOXP3 and ALOX15.

To determine whether FOXP3 itself was involved in the upregulation of ALOX15 during T_reg_ differentiation, we next incubated naïve CD4^+^ T-cells with TGF-β in the presence or absence of a FOXP3 inhibitor peptide and assessed ALOX15 expression. Here we found a significant inhibition of ALOX15 expression in cells incubated with the FOXP3 inhibitor, suggesting that FOXP3 regulated ALOX15 expression (Fig. 7E). Given that the *ALOX15* promoter expresses FOXP3 binding elements, we next tested whether FOXP3 directly regulated *ALOX15* promoter activity. For this purpose, we co-transfected HEK cells, that do not constitutively express this transcription factor (Supplementary Fig. S7C, D), with a vector expressing the *ALOX15* promoter region coupled to a luciferase reporter and either a vector expressing *FOXP3* or a control vector and assessed luciferase activity. These experiments demonstrated that FOXP3 upregulated the activity of the *ALOX15* promoter as assessed through an increase in luminescence signal in cells co-transfected with a *FOXP3* and the *ALOX15* promoter when compared with cells transfected with the *ALOX15* promoter and a control vector (Fig. 7F).

Since naive CD4^+^ T-cells express ALOX15 and inhibition of this enzyme regulated FOXP3 expression, we next questioned whether ALOX15-derived lipid mediator production is increased prior to FOXP3 upregulation during T_reg_ differentiation. Here we found that several ALOX15-derived mediators from the DHA and n-3 DPA bioactive metabolomes, that included RvD3, were upregulated during the early stages (after 1h) of T_reg_ differentiation (Fig. 7G, Supplementary Table S6). Of note, incubation of T_regs_ with RvD5_n-3 DPA_ or RvD3 rescued the expression of FOXP3 in T_regs_ incubated with an ALOX15 inhibitor (Fig. 7H). Together these findings unveil a previously unappreciated biological circuit whereby, ALOX15-derived mediators are increased early on during T_reg_ differentiation. These mediators, including RvD5_n-3 DPA_ and RvD3, in turn upregulate FOXP3 expression that then augments the ALOX15 expression (Fig. 7I).

## Discussion

In the present study we established a role for ALOX15 in T_reg_ differentiation and function. Expression and activity of this enzyme were upregulated during the early stages of T_reg_ differentiation leading to an increase in the levels of several mediators including the ALOX15-derived RvD5_n-3 DPA_ and RvD3. Inhibition of ALOX15 or deletion of this enzyme reduced the expression of FOXP3, altered cellular metabolic pathways and the transcriptome of T_regs_. This was linked with an impaired ability of these cells to exert their protective actions, including the modulation of Th1 responses and macrophage efferocytosis, as determined both in vitro and in vivo. These findings highlight a novel role for ALOX15 in governing fundamental cellular processes in the adaptive arm of the immune system by regulating T_reg_ differentiation and function.

In mammals there are two main T_reg_ subtypes that mature in distinct organs *via* site specific mechanisms [5]. The first subset is referred to as thymic T_regs_ that mature in the thymus from CD4^+^CD8^+^ cells *via* the engagement of TCR by agonist ligands in the presence of trophic cytokines. The second subset originates in peripheral tissues *via* the activation of conventional CD4^+^ cells by a host of agonists, including natural antigens from commensal bacteria [5]. A defining feature common to these T_reg_ subsets is the upregulation of the transcription factor FOXP3. Of note, deficiencies in *Foxp3* cause lymphoproliferation and multiorgan autoimmunity in Scurfy mutant mice and humans with immunodysregulation polyendocrinopathy enteropathy X-linked [28]. In the present studies, we found that inhibition of ALOX15 or deletion of the enzyme decreased the expression of FOXP3 in both in vitro generated T_regs_, and in vivo in splenic T_regs_, an observation that was linked with increased vascular lipid accumulation and *Tnf-a expression* in *Alox15*-deficient mice when fed a western diet.

Different T-cell subtypes rely on distinct metabolic pathways in order to exert their cellular functions [29]. T_regs_ utilize oxidative phosphorylation to regulate cellular responses and disruption in this pathway is linked with a loss in the ability of these cells to exert their regulatory actions [29, 30]. In the present study, we found that loss of ALOX15 activity or expression led to an alteration of cellular metabolism with an upregulation of glycolysis, as measured by an increase in the levels of several glycolytic products including lactate. These changes in cellular metabolism were coupled with a marked alteration in the transcriptome of cells deficient in *ALOX15*, including an upregulation of genes linked with several effector pathways, such as T-cell mediated cytotoxicity and responses to interferon.

Emerging evidence highlights a role of T_regs_ in the regulation of resolution processes, for example *via* the upregulation of macrophage efferocytosis [4]. Clearance of apoptotic cells by macrophages is a key step in the resolution of inflammation [14, 19, 20, 31]. Defects in this key process are implicated in the propagation of a number of chronic inflammatory conditions that include cardiovascular inflammation [14, 19, 20, 31]. In the present studies, we observed that T_regs_ expressed several SPM biosynthetic enzymes including ALOX5, ALOX12 and ALOX15. Intriguingly these enzymes are also involved in the production of pro-inflammatory mediators, for example ALOX5 produces the potent leukocyte chemoattractant LTB_4_ [8]. Recent studies demonstrate that, in addition to expression, subcellular localization of these enzymes contributes to their product profile, ie SPM vs pro-inflammatory eicosanoids [15, 18]. Since we observed that SPM formation was preferentially upregulated in T_regs_, our findings suggest that the subcellular localization of these enzymes is also potentially regulated during T_reg_ maturation. Furthermore, the formation of these mediators was critical in directing T_reg_ differentiation, since genetic deletion of *Alox15* or inhibition of ALOX15 activity during T_reg_ differentiation led to cells with an impaired ability to upregulate efferocytosis in monocyte-derived macrophages when tested both in vitro and in vivo. We also found that in *Alox15-*deficient mice fed a western diet there was a marked increase in vascular inflammation as measured by an increase in aortic lesions. This vascular inflammation was reversed when WT T_regs_ were adoptively transferred to *Alox15*-deficient mice.

Pro-resolving mediators are central in maintaining homeostasis and promoting the termination of inflammation and include the DHA and n-3 DPA-derived resolvins. Congruently, studies investigating mechanisms that are dysregulated during chronic inflammation found that production and/or actions of these protective molecules are altered in many experimental and human chronic inflammatory conditions [32–37]. Recent studies have raised the possibility that ALOX15 may also be important in the maintenance of tissue homeostasis as well as in regulating the differentiation of monocytes to macrophages [19, 38]. Findings made herein demonstrate RvD5_n-3 DPA_ and RvD3 play a central role in the differentiation of naive CD4^+^ T-cells to T_regs_. The upregulation of these molecules during the early stages of T_reg_ differentiation was observed to play a central role in their differentiation via the upregulation of FOXP3. This pivotal role for pro-resolving mediators in T_reg_ differentiation was further underscored by results obtained following either pharmacological inhibition or deletion of ALOX15, which led to cells that displayed blunted regulatory actions.

In summation, herein we found that an increase in ALOX15-derived lipid mediators antecedes the upregulation of FOXP3. This nuclear factor in turn upregulated the expression of ALOX15. Inhibition of ALOX15 activity or expression early on in the differentiation program led to cells that were markedly deficient in exerting their protective functions. These observations support a central role for ALOX15 in shaping the biological properties of T_regs_. As such, the present study uncovered a previously unappreciated role for this enzyme in the regulation of T_reg_ differentiation and, consequently function. These findings provide new insights into molecular and cellular mechanisms involved in the maintenance of homeostasis and the resolution of inflammation.

## Materials and methods

### Experimental model and subject details

#### Animal studies

10–12 week old, female and male, C57/Black6 (Charles River), *Alox15 deficient (*^−/−^*)* (The Jackson Laboratory, St. Bar Harbor, Maine, United States) and RAG 1 deficient *(*^−/−^*)* (The Jackson Laboratory) mice were used in the reported studies. The experiments strictly adhered to UK Home Office regulations (Guidance on the Operation of Animals, Scientific Procedures Act, 1986) and Laboratory Animal Science Association (LASA) Guidelines (Guiding Principles on Good Practice for Animal Welfare and Ethical Review Bodies, 3rd Edition, 2015). Animals were kept on a 12 h light dark cycle, with lights turned on at 7:00h and lights turned off at 19:00h under specific pathogen free housing and had access to food and water *ad libitum*. Animals were randomly assigned to control and experimental groups. The investigators were not blinded to group assignments.

#### Human primary cells

Healthy human peripheral blood mononuclear cells (PBMCs) were isolated from leukocyte cones obtained from the NHS Blood and Transplant bank. Experiments were conducted in accordance with a protocol approved by Queen Mary Research Ethics Committee (QMREC 2014:61) and in accordance with the Helsinki declaration. Leukocyte cones were unidentified, consequently no information was available on sex, age, their involvement in previous experiments or if they were drug or test naïve. The HL60 cell line was established in a female donor. The HEK293 cell line was established in human embryonic kidney.

### Method details

#### Isolation of naïve CD4^+^ T cells

PBMCs were isolated using published protocols [19]. Naïve CD4^+^ T-cells were isolated from PBMCs by immunomagnetic negative selection using the EasySep™ Human Naïve CD4^+^ T Cell Isolation Kit (StemCell Technologies, Vancouver, Canada) and following the manufacturer’s instructions.

Splenic cells were obtained following dissociation of spleens from WT and *Alox15*^−/−^ mice using a 70 µM filter. Mouse naïve CD4^+^ T-cells were then isolated from splenocytes by immunomagnetic negative selection using the EasySep™ Mouse Naïve CD4^+^ T Cell Isolation Kit (StemCell Technologies) and following the manufacturer’s instructions that yielded ~99% CD4^+^ T-cells.

#### T_reg_ cultures

T_regs_ were obtained using published protocols with a slight modification [1], whereby naïve CD4^+^ T-cells (Th0) were suspended in complete X-VIVO 15 medium (Lonza, Basel, Switzerland, 2 mM L-Glutamine, 100 U/ml penicillin (Sigma, St. Louis, Missouri, United States), 100 mg/ml streptomycin (Sigma), 10 mM HEPES (Gibco, Thermo Fisher Scientific, Waltham, Massachusetts, USA), supplemented with IL-2 (100 U/ml, Biolegend, San Diego, California, United States) and TGF-β (5 ng/ml, Biolegend). Dynabeads T-Activator CD3/CD28 (Gibco, Thermofisher Scientific) were added at a ratio of one bead per cell, and then incubated at 37 °C, 5 % CO_2_, for 7 days.

To determine the expression of lipid mediator biosynthetic enzymes, naive CD4^+^ T-cells (Th0) were incubated for 1, 3, 6, 18, 24, 48, 96 or 168 h as describe above. Cells were then collected for qPCR and flow cytometry analysis. To determine the expression of ALOX15 and FOXP3 during T_reg_ differentiation, naïve CD4^+^ T cells (Th0) were incubated for 0, 24, 48, 96 or 168 h as described above, and collected for flow cytometry analysis. To assess the lipid mediator profiles of Th0 and T_regs_, cells were isolated and cultured as detailed above in complete X-VIVO 15 medium without Phenol Red (Lonza), with T_regs_ differentiated as for 1 h, 24 h or 168 h prior to quenching in ice-cold methanol containing deuterium labeled internal standards. Samples were then held at −20 °C or below prior to lipid mediator profiling.

To assess human and mouse T_reg_ phenotype and for co-cultures of T_regs_ with Th1 or Th17 cells, Th0 were incubated at day 0 or 3, with an ALOX15 inhibitor (5 μM; PD146176, Cayman Chemical, Ann Arbor, Michigan, United States) or vehicle (0.01% DMSO) and then differentiated as described above for 7 days. In select experiments, T_regs_ were incubated with ALOX15 inhibitor or vehicle (30 min, 37 °C) and then with RvD5_n-3 DPA_ (1nM) or RvD3 (1nM) and differentiated for 7 days, with mediators supplemented every other day for the 7 day duration of the experiments. For RNA-seq analysis, Th0 from WT and Alox15^−/−^ mice were differentiated as described before for 2 days. The cells were then harvested, washed and resuspended in 350 μl of RLT Plus buffer (Qiagen, Hilden, Germany) and stored at −80 °C for analysis as described below.

To assess the lipid mediator profiles of human and mouse T_regs_ in presence or absence of ALOX15 activity, cells were isolated and cultured as detailed above in complete X-VIVO 15 medium without Phenol Red (Lonza). Mouse cells were isolated from WT and *Alox15*^*−/−*^ mice; human cells were incubated with an ALOX15 inhibitor (5 μM; PD146176, Cayman Chemical) or vehicle (0.01% DMSO) from day 0 and medium refreshed at day 3. Cells were differentiated for 7 days prior to quenching in ice-cold methanol containing deuterium labeled internal standards. Samples were then held at −20 °C or below prior to lipid mediator profiling.

#### Th1 and Th17 cultures

Th1 cells were obtained following published procedures with a slight modification [1], whereby incubating Th0 were suspended in complete X-VIVO 15 medium supplemented with IL-12 (10 ng/ml, Biolegend) and IL-2 (50 U/ml, Biolegend). Dynabeads T-Activator CD3/CD28 (Gibco, Thermofisher Scientific) were then added at a ratio of one bead per cell, and cells incubated at 37 °C, 5% CO_2_, for 7 days. Cellular phenotype was ascertained using flow cytometry and fluorescently labeled antibodies as detailed below.

Th17 cells were obtained in accordance with published protocols with a slight modification [1]. Th0 cells were suspended in complete X-VIVO 15 medium supplemented with IL1-β (10 ng/ml, Biolegend), IL-6 (20 ng/ml, Biolegend), IL-23 (10 ng/ml. Biolegend) and TGF-β (5 ng/ml, Biolegend). Dynabeads T-Activator CD3/CD28 (Gibco, Thermofisher Scientific) were then added at a ratio of one bead per cell, and cells then incubated at 37 °C, 5% CO_2_, for 7 days. Cellular phenotype was ascertained using flow cytometry and fluorescently labeled antibodies as detailed below.

#### Macrophage differentiation

Human monocyte-derived macrophages were prepared using published protocols [39]. Briefly, PBMCs were plated in a 10 cm low adhesion tissue culture plates and incubated at 37 °C for 30 min in Dulbecco’s Phosphate-Buffered Saline (DPBS, containing calcium and magnesium (DPBS^+/+^, Gibco). Non-adherent cells were then removed by adding DPBS^−/−^ and washing vigorously. After the last wash, complete RPMI 1640 (Gibco) containing 10% of human serum (Gibco), 100 U/ml penicillin (Sigma), 100 mg/ml streptomycin (Sigma) and 20 ng/mL GM-CSF (Biolegend), was added per plate. The cells were incubated for 7 days at 37 °C, 5% CO_2_.

Murine bone marrow-derived macrophages were prepared from bone marrow isolated from WT mice as previously described [19]. Briefly, bone marrows were obatined from both femurs and tibias using DPBS^−/−^. Cells were then seeded in a 10 cm low adhesion tissue culture plates and incubated in complete RPMI 1640 containing 10% of Fetal Bovine Serum (FBS, Gibco), 100 U/ml penicillin, 100 mg//ml streptomycin and 20 ng/mL GM-CSF.

#### T_regs_ functional assessment in vitro

##### Inhibition of T-cell proliferation

Naïve CD4^+^ T lymphocytes (CD4^+^CD25^−^, Effector T cells) were isolated as described above and stained with carboxyfluorescein succinimidyl ester (CFSE) dye. Briefly, cells were suspended at 10^7^ cells in a 3 μM CFSE solution and incubated for 8 min at room temperature (RT) in the dark with shaking. After incubation, an equal volume of FBS was added to neutralize the dye, DPBS containing calcium and magnesium (DPBS^+/+^) was added, and cells were centrifuged at 423 × *g* for 5 min. Cells were then seeded in 96 well plates in complete X-VIVO 15 medium at 37 °C, 5% CO_2_. These were then incubated with human or mouse T_regs_, prepared as described above, at a ratio of, 1:1, 0.5:1, 0.25:1 and 0:1, (T_reg_:T-effector) for 3 days. Cellular proliferation was then assessed using flow cytometry.

##### Suppression of Th1 or Th17 cytokine expression in T-effector cells

Naïve CD4^+^ T lymphocytes isolated from human PBMCs or mouse WT or *Alox15*^*−/−*^ splenocytes were differentiated as described above to Th1 and Th17 cells. Cells were then seeded in 96 well plates in complete X-VIVO 15 medium at 37 °C, 5% CO_2_. These cells were incubated with human or mouse T_regs_, prepared as described before, at a ratio of 0.5:1 or 0:1 (T_reg_:T-effector). The cells were then incubated with Dynabeads T-Activator CD3/CD28 (one bead/cell) and incubated for 24 h. Eighteen hours into the 24 h incubation, GolgiSTOP (BD Bioscience, Franklin Lakes, New Jersey, USA) was added and the expression of IFN-γ and IL-17A was assessed in Th1 and Th17 cells respectively using flow cytometry.

##### Macrophage efferocytosis

Macrophages, prepared as described above, were seeded in 96 well plate in complete RPMI 1640 medium at 37 °C, 5% CO_2_. These cells were then incubated with human T_regs_, prepared as described above, at a ratio of 1:4 (T_regs_: Macrophage). The cultures were stimulated with 62.5 ng/ml of CD3 antibody and incubated for 4 days as previously described [4], with a slight modification. Apoptotic HL60 were prepared and stained using PKH26 Red Fluorescent Cell Linker kit (Sigma) according to publish protocols [19]. After incubation, T_regs_ were washed with DPBS^−/−^ and fluorescently labeled apoptotic HL60 (1:3, macrophages to apoptotic HL60) were added to the macrophages, and efferocytosis was assessed using CellDiscoverer 7 Microscope (Zeiss, Oberkochen, Germany) for 1 h.

#### In vivo studies

##### In vivo efferocytosis

RAG1^−/−^ mice were administered zymosan (0.1 mg per mouse, *via i.p* injection) in 500 μl of DPBS^+/+^ (Gibco). After 30 h, 3 × 10^5^ of T_regs_ from WT or *Alox15*^−/−^ mice, prepared as described above, were injected intraperitoneally in accordance with published methods [4]. Four days after, 6 × 10^6^ PKH67-labeled apoptotic HL60 were injected intraperitoneally. One hour later peritoneal lavages were collected and incubated with an APC-Cy7 anti-mouse F4/80 antibody (Clone BM8, Biolegend) as detailed below, and efferocytosis in F4/80 positive macrophages was evaluated using flow cytometry and ImageStream (Amnis ImageStream X MK2, Merck Millipore, Watford, UK).

##### Vascular inflammation

WT and Alox15^−/−^ mice were fed a western diet for 8 weeks. At the end of the experiment aortas, blood and spleens were collected. The lesions in the aortic arch were determined by Oil Red O staining and quantified using Image J software as previously described [38]. Effector T cell phenotypes in the blood and spleen were analyzed using flow cytometry.

In select experiments, T_regs_ were obtained from WT and *Alox15*^*−/−*^ mice as described above, and were injected (3 × 10^5^ cells) via intravenously injection to *Alox15*^*−/−*^ mice. Mice were then fed with Western Diet for 8 weeks. At the end of the experiments, on week 8, aortas, blood and spleens were collected and aortic lesions together with T-cell phenotype determined as detailed above.

#### RNA extraction

Cell pellets and aortas were snap-frozen and stored at −80 °C until processing. For total RNA extraction, we used the QIAGEN RNeasy Plus Micro kit (Qiagen) or Plus Mini kit (Qiagen) following the protocol outlined in the QIAGEN RNeasy Plus Micro Hanbook 12/2014 or Plus Mini Hanbook 09/2020. Briefly, 350 µL Buffer RLT Plus (Qiagen) was added per cell pellet and aortic tissue was placed in tubes containing homogenizing beads and 600 µL Buffer RLT Plus (Qiagen). The cells were mixed by pipetting and the tissue was homogenized using a bead beater (Precellys 24 Tissue Homogenizer) for 30 s at 5500 rpm, and then placed on ice. Aortic lysates were then centrifuged at 10,000 × *g* for 5 min at room temperature. The samples were pipetted directly into a QIAshredder spin column. The genomic DNA (gDNA) was then removed by transferring the homogenized lysate to a gDNA Eliminator spin column. Then 1 volume of ethanol was added to the flow-through and the resultant solution mixed. The samples were then transferred to a RNeasy MinElute spin column to extract and purify the RNA. Extracted RNA samples were assessed for quantity and quality using the NanoDrop 8 000 spectrophotometer V2.0 (ThermoScientific, USA).

#### Real-time PCR

Total RNA was reverse-transcribed to cDNA using sequence specific primers for each enzyme analyzed (Quantitec primers assay, Qiagen) or Oligo (dT) 20 primers for aortic tissue samples (Invitrogen) in accordance with the SuperScript III Reverse Transcriptase protocol (Invitrogen, Carlsbad, California, USA). Real-time PCR reactions were performed using a QuantStudio 7 Flex System (Applied Biosystems, Foster City, California, USA) or a StepOne Plus Real-Time PCR System (Applied Biosystems) using PowerUp™ SYBR™ Green Master Mix (Applied Biosystems) following manufacturer’s instructions. Beta-Actin and RRN18S were used as reference genes. Data analysis was carried out using QuantStudio Real-time PCR software version 1.3 (Applied Biosystems) or StepOne Software v2.3 (Applied Biosystems). Relative expression was represented as change fold from Th0 (2^−ΔΔC^_T_) for enzyme expression, WT or KO mice that received KO cells for the vascular inflammation study.

#### Cholesterol measurement in plasma - HDL and LDL/VLDL fraction

Whole blood from WT and Alox15^−/−^ mice, fed WD and used in the passive transfer of T_regs_, was collected by using heparin-lined syringes *via* cardiac puncture. Then, the blood was centrifuged at 2000 × *g* for 10 min at room temperature. The plasma was transfer to new tubes and storage at −80 °C until further use. For measurement of HDL and LDL/VLDL fractions in the plasma, we used the Cholesterol Assay Kit - HDL and LDL/VLDL (Abcam, ab65390). Briefly, to separate HDL and LDL/VLDL, 90 μl of the samples were mixed with 90 μl of precipitation buffer and incubated for 10 min, room temperature. The samples were then centrifuged for 10 min at 2000 × *g*. The supernatant, HDL fraction, was transferred to a new tube and the centrifugation was repeated. The fraction pellet, LDL/VLDL, was resuspended with PBS. Samples and standards were incubated with total cholesterol reaction mix (with esterase) for 60 min at 37 °C. The fluorescence was measure in the NOVOstar 700-0107 Plate reader (Ex/Em = 538/587 nm).

#### Lipid mediator profiling

Cells were harvested for LC-MS-MS-based profiling and extracted using solid-phase extraction columns as in [40, 41]). Briefly, 2V of ice-cold methanol containing deuterium labeled internal standards, representing each region in the chromatographic analysis (500 pg each), was added to each sample to facilitate identification and quantification. Samples were then extracted using solid-phase (C-18) extraction columns as in [19]. Samples were injected on a Shimadzu LC-20AD HPLC and a Shimadzu SIL-20AC autoinjector, paired with a QTrap 6500+ (Sciex, Framingham, Massachusetts, USA) following method described in [19]. Each LM was identified using established criteria including matching retention time to synthetic and authentic standards, an area under the curve greater than 2000 units and at least 4 data points in the peak. Calibration curves were obtained for each using synthetic compound using a blank and standard mixtures of each of the molecules at 0.78, 1.56, 3.12, 6.25, 12.5, 25, 50, 100, and 200 pg that gave linear calibration curves with *r*^2^ values of 0.98–0.99.

#### Transcriptomic analysis of WT and Alox15^−/−^ T_regs_

##### RNA extraction, library preparation and sequencing

Cells were prepared as detailed above and samples preserved in RLT Plus Buffer (Qiagen) at −80 °C until analysis. The MACE-Seq Kit analysis was conducted by GenXPro GmbH (Goethe University, Frankfurt, Germany). Here, RNA was extracted using Zymo IC columns (Zymo, California, USA), following manufacturer’s instructions. Contaminating DNA was then removed using DNAseI (NEB, Ipswich, Massachusetts, USA) and the RNA was purified using the Zymo RNA Clean & Concentrator Kit (Zymo). MACE-Seq libraries from the extracted RNA were then prepared using the MACE-Seq Kit (GenXPro) according to the manufacturer’s instructions. The resultant samples were then sequenced on an Illumina NextSeq500 machine with at least 5 M reads per sample respectively (GEO Accession Number: GSE140750).

#### Glycolytic and Tricarboxilic acid (TCA) cycle profiling

Samples were placed in 1V ice cold MeOH containing deuterated internal standards (^13^C_2_-Citrate, ^13^C_2_-Fumarate and ^13^C_6_-Glucose, 5 μg, 5 μg and 10 μg, respectively). 1V of ice cold water was added followed by the addition of 2V of Chloroform. Samples were then centrifuged for 10 min at 4000 × g. Aqueous phase was collected and brought to dryness under a gentle nitrogen stream using TurboVap LV prior suspension in H_2_O for LC-MS/MS profiling.

Extracted samples were analyzed using a Qtrap 6500+ (AB Sciex) equipped with a Shimadzu SIL-20AC autoinjector and LC-20AD binary pump (Shimadzu Corp, Kyoto, Japan). Separation was conducted with a method adapted from [42]. Briefly, a Synergi Hydro-RP column (250 × 4.6 mm ×4 μm, Phenomenex) column, maintained at 30 °C, was used with a gradient of methanol/water/acetic acid of 0:100:0.5 (vol:vol:vol) that was ramped to 100:0:0.5 (vol:vol:vol) over 16 min The flow rate was maintained at 0.5 ml/min. To monitor and quantify the levels target molecules, a multiple reaction monitoring (MRM) method was adapted from [43] (Supplementary Table S7). Quantitation was achieved using standard curves for each of the compounds of interest.

#### ImageStream

For ImageStream analysis, cells were stained for surface markers for 30 min at 4 °C, in FACS Buffer (DPBS^−/−^ +0.02% bovine serum albumin, BSA). For intracellular staining, cells were fixed with Fix/Perm Buffer (diluted 1:4, eBioscience, San Diego, California, USA) for 20 min at RT, and then washed twice with FACS Buffer for 5 min at 1328 × *g*. Cells were permeabilized using Fc block/Perm Buffer (diluted 1:2, in 1x Permeabilization buffer, eBioscience) for 30 min at RT, and then stained for intracellular markers for 30 min at 4 °C, in Perm Buffer (eBioscience). Staining was assessed using ImageStream X MK2 and analysis was performed using IDEAS® (Image Data Exploration and Analysis Software, Version 6.0). In selected experiments to assess efferocytosis by peritoneal macrophages from RAG1^−/−^, cells were stained for surface markers with the following antibodies: APC-Cy7 anti-mouse F4/80 (Clone BM8, Biolegend) and PE anti-mouse CD64 (Clone, Biolegend). For the experiments evaluating biosynthetic enzymes, cells were stained for intracellular markers with the following antibodies: Alexa Fluor 647 anti-15-LOX type 1 (Bioss Antibodies, Woburn, Massachusetts, USA), Alexa Fluor 488 anti-COX-2 (Clone D5H5, Cell Signaling, London, UK), Dylight 405 anti-5-LOX (Novus Biologicals, Littleton, Colorado, USA) and Alexa Fluor 594 anti-12-LOX (Abgent, San Diego, California, USA).

#### p38, ERK1/2 and FOXP3 inhibition

##### p-38 and ERK1/2 inhibition

Naïve CD4 T Cells, prepared as described above, were seeded in 96 well plates in complete X-VIVO 15 supplemented with or without TGF-β (5 ng/ml) and Dynabeads T-Activator CD3/CD28 (one bead/cell). Before supplementation, cells were incubated for 30 min with ERK1/2 inhibitor (SCH 772984, Cayman) or p-38 inhibitor (SB202190, Cayman) at 2.5 μM and 5 μM. To determine SMAD2/3, STAT1 and STAT3 phosphorylation cells were harvested after 12 h and phosphorylation status determined using flow cytometry. Assessment of FOXP3 and ALOX15 expression conducted after 24 h using flow cytometry.

##### FOXP3 inhibition

Naïve CD4 T Cells, prepared as described above, were seeded in 96 well plates in complete X-VIVO 15 supplemented with TGF-β (5 ng/ml, Biolegend) and Dynabeads T-Activator CD3/CD28 (one bead/cell, Gibco), and in the presence of FOXP3 inhibitor peptide (100 uM, P60 peptide, Abbiotec, Escondido, California, USA) or vehicle (0.01% DMSO). ALOX15 expression was assessed after 7 days by flow cytometry.

#### Flow cytometry

For Flow cytometry analysis, cells were stained with Aqua Zombie Live/Dead (dilution 1:1 000 in DPBS, Biolegend) for 30 min on ice, and then stained for surface markers for 30 min at 4 °C, in FACS Buffer. For intracellular staining, cells were fixed with Fix/Perm Buffer (diluted 1:4, eBioscience) for 20 min at RT. Cells were then permeabilized with Fc block/Perm Buffer (diluted 1:2 in 1x Permeabilization buffer, eBioscience) for 30 min at RT, and stained for intracellular markers for 30 min at 4 °C, in Perm Buffer (eBioscience). The samples were then evaluated using LSRFortessa cell analyzer (BD Biosciences) and analyzed using FlowJo software (Tree Star Inc., V10).

In selected experiments to evaluate the biosynthetic enzymes, cells were stained for intracellular markers with the following antibodies: Alexa Fluor 647 anti-15-LOX type 1 (Bioss Antibodies; Cat no: bs-6505R-A647), Alexa Fluor 488 anti-COX-2 (Clone D5H5, Cell Signaling Technology Cat no: 13596S), Dylight 405 anti-5-LOX (Novus Biologicals, Cat no: NB110-58748AF405) and Alexa Fluor 594 anti-12-LOX (Abgent Cat no: AP8877B).

For the analysis of human and mouse T_reg_ phenotype, for the surface staining was used the following antibodies: ***human*** – APC-Cy7 anti-human CD3 (Clone SK7, Biolegend; Cat no: 344818), PerCP-Cy5.5 anti-human CD4 (Clone RPA-T4, Biolegend; Cat No: 300530), Brilliant Violet 488 anti-human CD25 (Clone BC96, Biolegend; Cat no: 302616), Brilliant Violet 711 anti-human CD127 (Clone A019D5, Biolegend; Cat no: 351328); ***mouse*** - APC-Cy7 anti-mouse CD3 (Clone 17A2, Biolegend; Cat no: 100222), AF700 anti-mouse CD4 (Clone GK1.5, Biolegend; Cat no: 100430), Brilliant Violet 785 anti-mouse CD25 (Clone PC61, Biolegend; Cat no:102051), FITC anti-mouse CD127 (Clone A7R34, Biolegend; Cat no: 135008), Brilliant Violet 650 anti-mouse CD127 (clone A7R34, Biolegend; Cat no: 135043). For the intracellular staining the following antibodies were used: ***human*** - Brilliant Violet 421 anti-human FOXP3 (Clone 206D, Biolegend; Cat no: 320124), PE anti-human FOXP3 (Clone 206D, Biolegend; Cat no: 320107), PE-Cy7 anti-human/mouse T-bet (Clone 4B10, Biolegend; Cat no: 644824), Alexa Fluor 647 anti-human RORγt (Clone Q21-559, eBioscience; Cat no: 563620); ***mouse*** - Brilliant Violet 421 anti-mouse FOXP3 (Clone MF-14, Biolegend; Cat no: 126419), PE anti-mouse FOXP3 (Clone MF-14, Biolegend), PE-Cy7 anti-human/mouse T-bet (Clone 4B10, Biolegend; Cat no: 644824), PE anti-mouse RORγt (Clone B2D, Biolegend; Cat no: 12-6981-82).

For the analysis of T_regs_ incubations with Th1 and Th17, the surface staining was performed using the antibodies described above with the exception of Brilliant Violet 488 anti-human CD25 (Clone BC96, Biolegend; Cat no: 302638), here it was used Brilliant Violet 785 anti-human CD25 (Clone BC96, Biolegend; Cat no: 302616). For the intracellular staining, in addition to the antibodies described above we used ***for human cells*** - APC anti-human IFN-γ (Clone B27, Biolegend; Cat no: 506510), PE anti-human IL-17A (Clone BL168, Biolegend; Cat no: 512306), Alexa Fluor 488 anti-human RORγt (Clone Q21-559, eBioscience; Cat no: 563621) instead of Alexa Fluor 647 anti-human RORγt (Clone Q21-559, eBioscience; Cat no: 563620); ***for mouse cells*** - APC anti-mouse IFN-γ (Clone XMG1.2, Biolegend; Cat no: 506510), Brilliant Violet 650 anti-mouse IL-17A (Clone TC11-18H10.1, Biolegend; Cat no: 506929).

For the experiments assessing the phosphorylation of target kinases, cells were fixed with paraformaldehyde (PFA) 4% in DPBS^+/+^ for 20 min at RT and permeabilized with ice-cold methanol for 30 min at 4 °C. Cells were incubated with anti-p-Smad2 (Ser465/467)/p-Smad3 (Ser423/425) (Clone D27F4, Cell Signaling; Cat no: 8828S), anti- p-Stat1 (Ser727) (Clone D3B7, Cell Signaling; Cat no: 8826S) p-Stat3 (Ser727) (Clone D8C2Z, Cell Signaling; Cat no: 94994S) for 1 h at RT. Cells were then incubated with a secondary antibody, Alexa Fluor 594 anti-rabbit IgG (Clone Poly4064, Biolegend; Cat no: 406418), for 30 min at RT.

To assess the ability of T_regs_ to regulate macrophage phenotype in vivo cells were collected from the peritoneum of RAG^−/−^ mice after WT and Alox15^−/−^ T_regs_ transfer as detailed above. These were then stained for surface markers using the following antibodies: APC-Cy7 anti-mouse F4/80 (Clone BM8, Biolegend; Cat no: 123117), Brilliant Violet 650 anti-mouse I-A/I-E (MHC II) (Clone M5/114.15.2, Biolegend; Cat no: 107641), PE-Cy5 anti-mouse/human CD11b (Clone M1/70, Biolegend; Cat no: 101210), PerCP-eFluor710 anti-mouse TIM-4 (Clone 54 RMT4-54, eBiosciences; Cat no: 46-5866-28), PE anti-mouse CD64 (FCγRI) (Clone X54-5/7.1, Biolegend; Cat no: 139303), For the intracellular staining, the following antibodies were used: PE-Dazzle 594 anti-mouse IL-10 (Clone JES5-16E3, Biolegend; Cat no: 505034), Brilliant Violet 421 anti-mouse TGF-β1 (Clone TW7-16B4, Biolegend; Cat no: 141407), PE anti-mouse Arginase 1 (R&D; Cat no: IC5868P), Alexa Fluor 647 anti-human iNOS (Clone 4E5, Novus; Cat no: NBP2-22119AF647) and protein expression was then evaluated as above.

#### Amplification of FOXP3 plasmid

##### Transformation of Stbl3 chemically competent E.coli

One Shot Stbl3 chemically competent cells (ThermoFisher Scientific) were thaw and 1 µL of Foxp3 Lentiviral Vector (pLenti-GIII-CMV, ABM) or pLenti-III-Blank Vector (ABM) was added. Content of the vials were gently mixed and incubated on ice for 30 min The cells were then heat-shocked for 45 s at 42 °C (water bath) without shaking and placed on ice for 2 min After that 250 µL of pre-warmed SOC Medium (ThermoFisher Scientific) was added to each vial. They were then incubated at 37 °C for 1 h at whilst shaking at 225 rpm in a shaking incubator. The content of each vial was spread at several dilutions in kanamycin selective agar plates (50 µg/ml, ABM). These were then incubated at 37 °C for 16 h.

##### Growing cells expressing plasmid & plasmid amplification

One isolated colony was selected and inoculated in 10 mL of LB broth supplemented with 50 µg/ml of kanamycin. Cells were grown for 16−18 h in a shaking incubator at 37 °C, 240 rpm. For the plasmid amplification, 10 µL of the previous cell suspension was added to 100 mL of LB broth supplemented with 50 µg/ml of kanamycin and then placed in a shaking incubator at 37 °C, 180 rpm for 16–18 h.

##### Isolation of plasmid

Cells were pelleted at 4000 × *g* for 10 min at 4 °C. Plasmid isolation was performed by a ZymoPURE II Plasmid Midiprep kit, following the manufacturer’s instructions (Zymo Research, D4200).

#### Luminescence assay

*ALOX15* promoter activation by FOXP3 was assessed by co-transfection of HEK 293 cells with FOXP3 plasmid (Abm by NBS Biologicals, Huntingdon, Cambridgeshire, UK) and *ALOX15* promoter tagged with a luciferase reporter gene (Switchgear genomics, Carlsbad, California, USA). Briefly, HEK cells (ATCC) were resuspended in DMEM (Gibco/Invitrogen) supplemented with 10% FBS (Gibco), 2 mM L-Glutamine (Sigma), 100 units/ml penicillin + 100 μg/ml streptomycin (Sigma), seeded in a 96 well plate and transferred to an incubator at 37 °C, 5% CO_2_ for 18/24 h. They were then co-transfected with FOXP3 or Control plasmid + *ALOX15* promoter (ratio of 5:1) for 24 h, according to manufacturer’s instructions using Lipofectamine LTX & PLUS™ Reagent (Invitrogen). To assess *ALOX15* promoter activity in the transfected cells the luciferase substrate was added and the cells were incubated for 30 min at RT, according to manufacturer’s instructions (LightSwitch™ Luciferase Assay Reagent, Cat. Number: LS010). After incubation, BMG Labtech NOVOstar MicroPlate Reader was used to measure luminescence.

#### Statistics

Statistical analyses and data derivation were performed using R [44], Prism 8 or Microsoft Excel. All results were expressed as mean±s.e.m. Normality and equal distribution of variance between the different groups were analyzed using Shapiro–Wilk test. Differences between groups were assessed using one-sample *t* test or Wilcoxon test (normalized data), Unpaired *t* test with Welch’s correction or Mann–Whitney (2 groups), 1-way ANOVA or Kruskal–Wallis test (multiple groups) followed by post hoc Tukey’s test or Dunn’s test, 2-way ANOVA followed by post hoc Tukey’s test (between groups that have been split on two independent variables). Investigators were not blinded to group allocation or outcome assessment. The criterion for statistical significance was *p* ≤ 0.05. Sample sizes for each experiment were determined on the variability observed in preliminary experiments. Partial least squares-discrimination analysis (PLS-DA) was conducted using MetaboAnalyst 4.0 [45]. PLS-DA is based on a linear multivariate model that identifies variables that contribute to class separation of observations on the basis of their variables (expression of macrophage phenotypic markers). During classification, observations were projected onto their respective class model. The score plot illustrates the systematic clusters among the observations (closer plots presenting higher similarity in the data matrix).

##### Differential gene expression analysis

For differential gene expression analysis (*Alox15*^*−/−*^ T_regs_ *vs* WT T_regs_), good gene expression was considered if two or more counts per each million mapped reads were identified per sample, in at least four of the samples. EgdeR (Bioconductor R package; https://bioconductor.org/packages/release/bioc/html/edgeR.html) were used to calculate differential gene expression (as the fold-change) between the two groups. Statistical significance was considered with an adjusted (Benjamini–Hochberg procedure correction) *p* value < 0.05.

##### GO and pathway enrichment analysis

GO enrichment analysis was completed using the Bioconductor R package “TopGO” (https://bioconductor.org/packages/release/bioc/html/topGO.html)). The Kolmogorov–Smirnov (KS) test was used with a *p* value of 0.05 as threshold after Benjamini–Hochberg correction for the statistical significance enriched GO terms for the GO categories: biological processes, cellular component and molecular function. All the genes from *mus musculus* (GRCm38.p6) with their associated GO terms were obtained using the BioMart tool from Ens.e.m.bl [46]. Density plots for each enriched GO term were generated to identify the enriched GO term associated with the differentially expressed genes between the KO and WT groups.

Pathway analysis was performed uploading the differentially expressed genes in STRING [47] and identifying the enriched pathways from KEGG [48] (*p* value < 0.05, Fisher exact test followed by multiple comparison correction using Benjamini–Hochberg procedure) and Reactome [49] (*p* value < 0.05, hypergeometric distribution test with Benjamini–Hochberg procedure) databases. To create the enriched pathways figure, the STRING results were uploaded to Cytoscape 3.7.2 to highlight the different enriched pathways and differentially expressed genes.

## Supplementary information

Supplemental Figure legends

Supplemental Figures

Supplemental Tables

## Data Availability

The datasets used and/or analyzed during the current study are available from the corresponding author on reasonable request.
