# Supplementary material for: Loss of 15-lipoxygenase disrupts Treg differentiation altering their pro-resolving functions
Source: Cell Death Differ. 2021 May 27;28(11):3140–60. doi: 10.1038/s41418-021-00807-x (PMC8563763; doi:10.1038/s41418-021-00807-x)
Supplement: Supplementary file 1 — Supplemental Figure legends [file 41418_2021_807_MOESM1_ESM.docx]

**Supplemental Information**

**Figure S1. Gating strategy used to identify human T-cell subset. (A)** Representative dot plots depicting the isotype control antibodies for each of the antibodies used to identify T_regs_, **(B)** Representative dot plots depicting the gating strategy used to identify T_regs_. **(C-D)** Gating strategy used to define Th1 phenotype. **(C)** Representative dot plots depicting the isotype control antibodies for each of the antibodies used to identify Th1 cells, **(D)** Representative dot plots depicting the gating strategy used to identify Th1 cells. **(E-F)** Gating strategy used to define Th17 phenotype. **(E)** Representative dot plots depicting the isotype control antibodies for each of the antibodies used to identify Th17 cells, **(F)** Representative dot plots depicting the gating strategy used to identify Th17 cells. **Related to Figure 3, Figure 5, Figure S3.**

**Figure S2. Mouse Tregs express LOX and COX enzymes. (A)** Representative dot plots depicting the isotype control antibodies for each of the antibodies used to identify T_regs_, **(B)** Representative dot plots depicting the gating strategy used to identify T_regs_. (**C**) Expression of LOX and COX enzymes in murine T_regs_. Results are representative of n = 3-4 mice per group. **Related to Figure 1, Figure 3, Figure 5, Figure S3.**

**Figure S3. Inhibition of ALOX15 activity during T_reg_ differentiation shift the metabolic profile and reduces their ability to regulate Th17 responses. (A)** T_regs_ were obtained as detailed above, in the presence or absence of ALOX15 inhibitor (5µM), and the concentration of metabolites from the glycolysis and TCA pathways was determined using liquid chromatography-tandem mass spectrometry. Results are expressed as the fold change of metabolites identified in T_regs_ differentiated in the presence of ALOX15 inhibitor versus T_regs_ differentiated with vehicle alone. Red illustrates metabolites that were upregulated in *Alox15^-/-^* T_regs_ and blue metabolites that were downregulated in these cells. For metabolites denoted in white circles no differences were found between the two groups whilst those denoted in grey were not measured. Results are representative of n=10 donors per group from two distinct experiments. **(B,C)** Th17 cells were differentiated from CD4^+^ T lymphocytes (see methods for details) and incubated without or with T_regs_ (1:0,5 ratio) for 18h, after which GolgiSTOP was added and IL-17A expression determined 6h later using flow cytometry. Reduction in the frequency of IL-17A expressing cells (left panel) and in the expression of IL-17A per cell (right panel) for **(B)** human and **(C)** mouse Th17 cells. Results are mean ± s.e.m. of % change from control (vehicle). n=8 donors for human cells and 6 mice for mouse cells from at least 2 distinct experiments. For A **p<0.01, ****p<0.0001, One sample t test or Unpaired t test with Welch's correction; For B *p<0.05, ***p<0.001, using One sample t test or Unpaired t test with Welch's correction. **Related to Figures 4 and 5.**

**Figure S4. Lipid mediators are upregulated 24 hours into the T_reg_ differentiation program.** Naïve CD4^+^ T lymphocytes (Th0) were obtained from healthy volunteers and either placed in ice-cold methanol containing deuterium labeled internal standards or incubated with a T_reg_ differentiation cocktail. After 24 hours cells were placed in ice-cold methanol containing deuterium labeled internal standards. Lipid mediators were then extracted, identified and profiled using LC-MS/MS–based lipid mediator profiling (see methods for details). Pathway analysis for the differential expression of mediators from the (left panel) DHA and n-3 DPA, and (right panel) EPA and AA bioactive metabolomes in cells incubated with a T_reg_ differentiation complex when compared to naive CD4^+^ T-cells. Statistical differences between the concentrations (expressed as the fold change) of the lipid mediators from the cells incubated with the T_regs_ and Th0 cells were determined using a student Mann-Whitney test followed by a multiple comparison correction using Benjamini-Hochberg procedure. Results are representative of n=4 donors from two distinct experiments. **Related to Figure 5.**

**Figure S5. Differential ability of *Alox15^-/-^* T_regs_ to regulate peritoneal macrophage phenotype compared with WT T_regs_.** WT and *Alox15^-/-^* naive CD4^+^ T-cells were differentiated to T_regs_ (see methods for details) from. Peritonitis was initiated in RAG^-/-^ mice with 0.1mg of zymosan administered via i.p injection. After 30 hours, 3x10^5^ WT or *Alox15^-/-^* T_regs_ were injected i.p. Four days later 6x10^6^ PKH67-labelled apoptotic cells were injected i.p., after one-hour peritoneal macrophages were collected and macrophage phenotype was determined in **(A)** large peritoneal macrophages **(B)** small peritoneal macrophages using flow cytometry and PLS-DA analysis. Results are representative of n=8 mice per group. **Related to Figure 6.**

**Figure S6. Adoptive transfer of WT T_regs_ regulates T_effector_ phenotype in *Alox15^-/-^* mice administered a western diet.** WT and *Alox15^-/-^* mice were fed Western Diet for 8 weeks, **(A)** Blood and **(B)** spleen were collected and T_effectors_ activation was determined using fluorescently labeled Abs and flow cytometry. Results are mean ± s.e.m. of % change from WT cells, n=4 per group. *p<0.05, using one sample t test. (**C-D**) WT and *Alox15^-/-^* T_regs_ were differentiated from CD4^+^ T-cells then transferred to *Alox15^-/-^* mice (3x10^5^ cells per mouse) via i.v. injection. Mice were then fed Western Diet for 8 weeks. **(C)** Blood and **(D)** spleen were collected and T_effectors_ activation was determined using fluorescently labeled Abs and flow cytometry. Results are mean ± s.e.m. of % change from *Alox15^-/-^* mice that received *Alox15^-/-^* T_regs_, n=4 mice per group. *p<0.05, using one sample t test. **Related Figure 6.**

**Figure S7. ERK signaling pathway does not regulate ALOX15 expression. (A-B):** CD4^+^ T-cells were isolated from healthy volunteers and incubated with an ERK1/2 inhibitor (SCH772984 - 30 minutes) or vehicle (0.01% DMSO), then differentiated into T_regs_ for 24 hours (*see methods for details*). **(A)** FOXP3 **(B)** ALOX15 expression was determined using flow cytometry. Results are mean ± s.e.m of % change from TGF-β+CD3/CD28 stimulated cells; n=4-6 healthy volunteers; *p<0.05, ***p<0.001, One sample t test. **(C-D):** HEK 293 cells were either left transfected (NT) or transfected with a control plasmid (Ctrl) and a plasmid containing FOXP3 (FOXP3) for 24 hours and FOXP3 expression was determined using flow cytometry. **(C)** Amount of FOXP3 expressing cells, **(D)** levels of FOXP3 per cell. n=6 replicates from 2 independent experiments. *, p<0.05, **, p<0.01, determined using Kruskal-Wallis test and Dunn’s test for multiple comparisons. **Related Figure 7.**
