# Supplementary figures and images for: Loss of 15-lipoxygenase disrupts Treg differentiation altering their pro-resolving functions
Source: Cell Death Differ. 2021 May 27;28(11):3140–60. doi: 10.1038/s41418-021-00807-x (PMC8563763; doi:10.1038/s41418-021-00807-x)

## Supplemental Figures

Figure S1

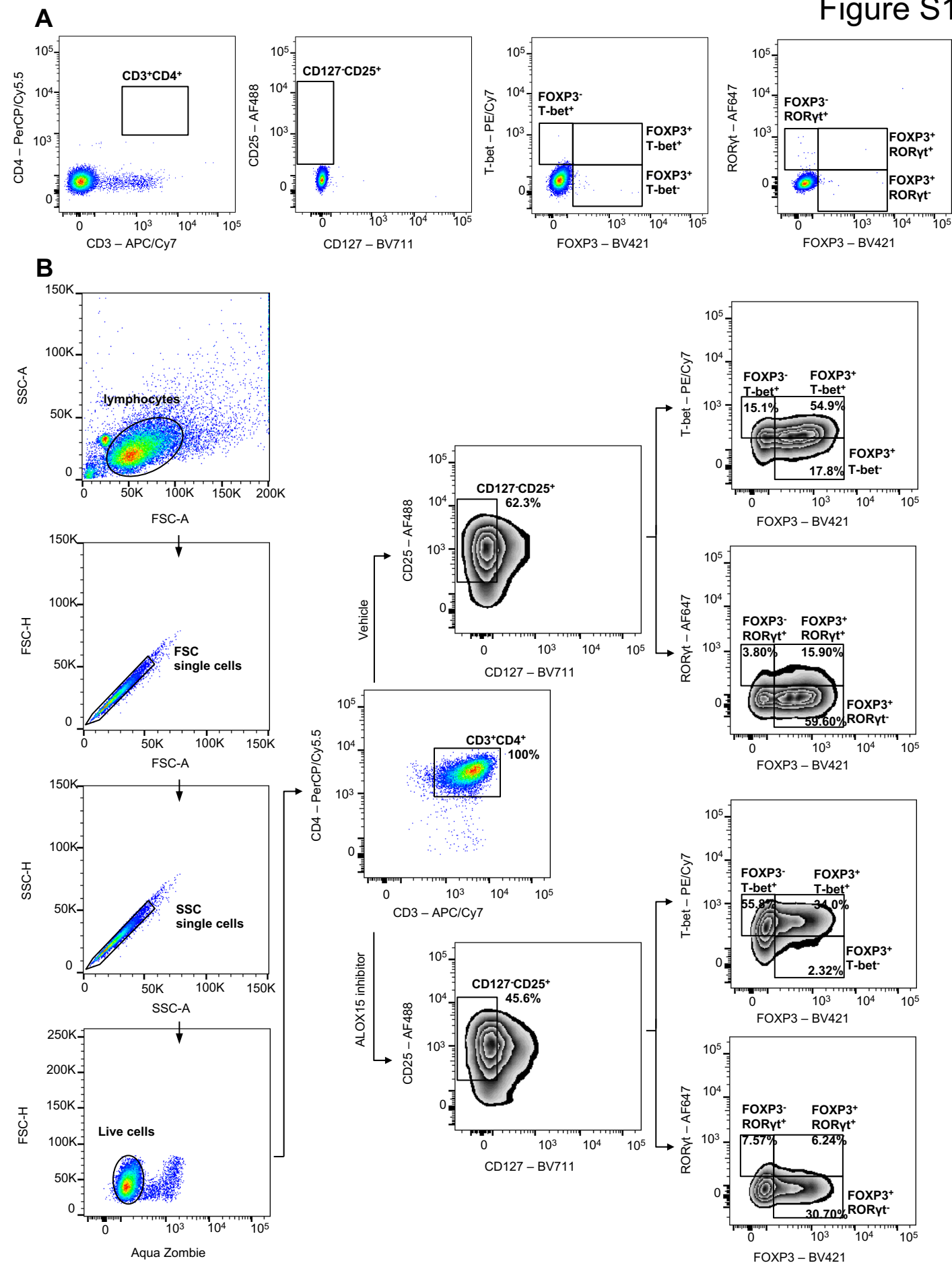

Figure S1

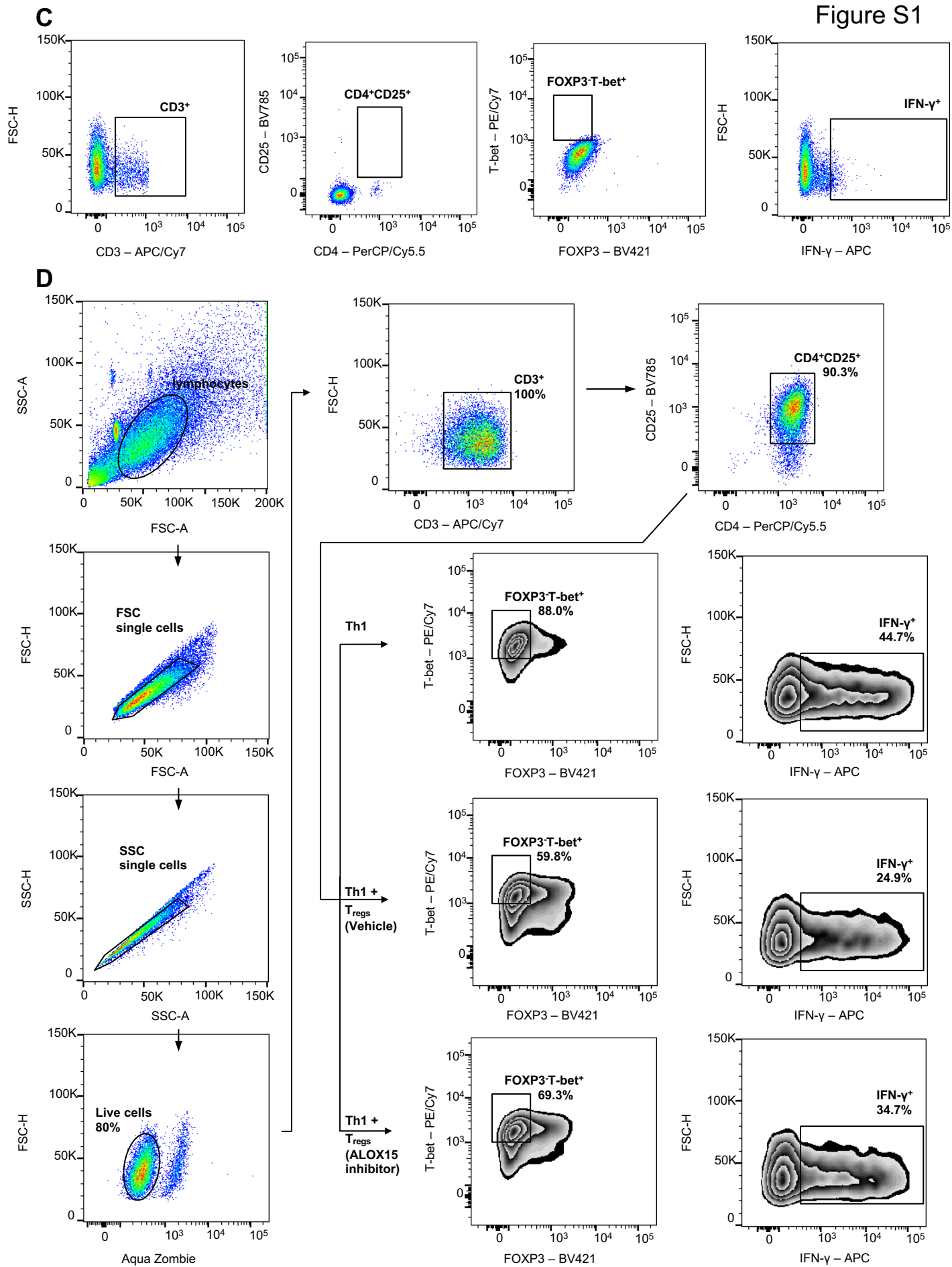

**E**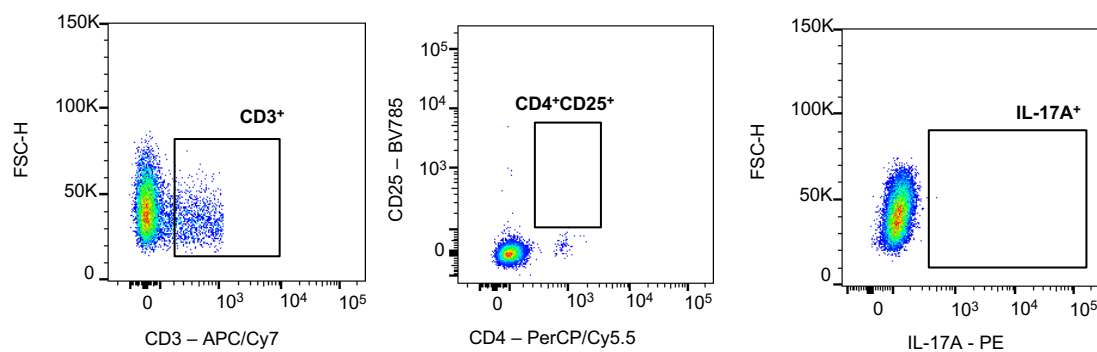**F**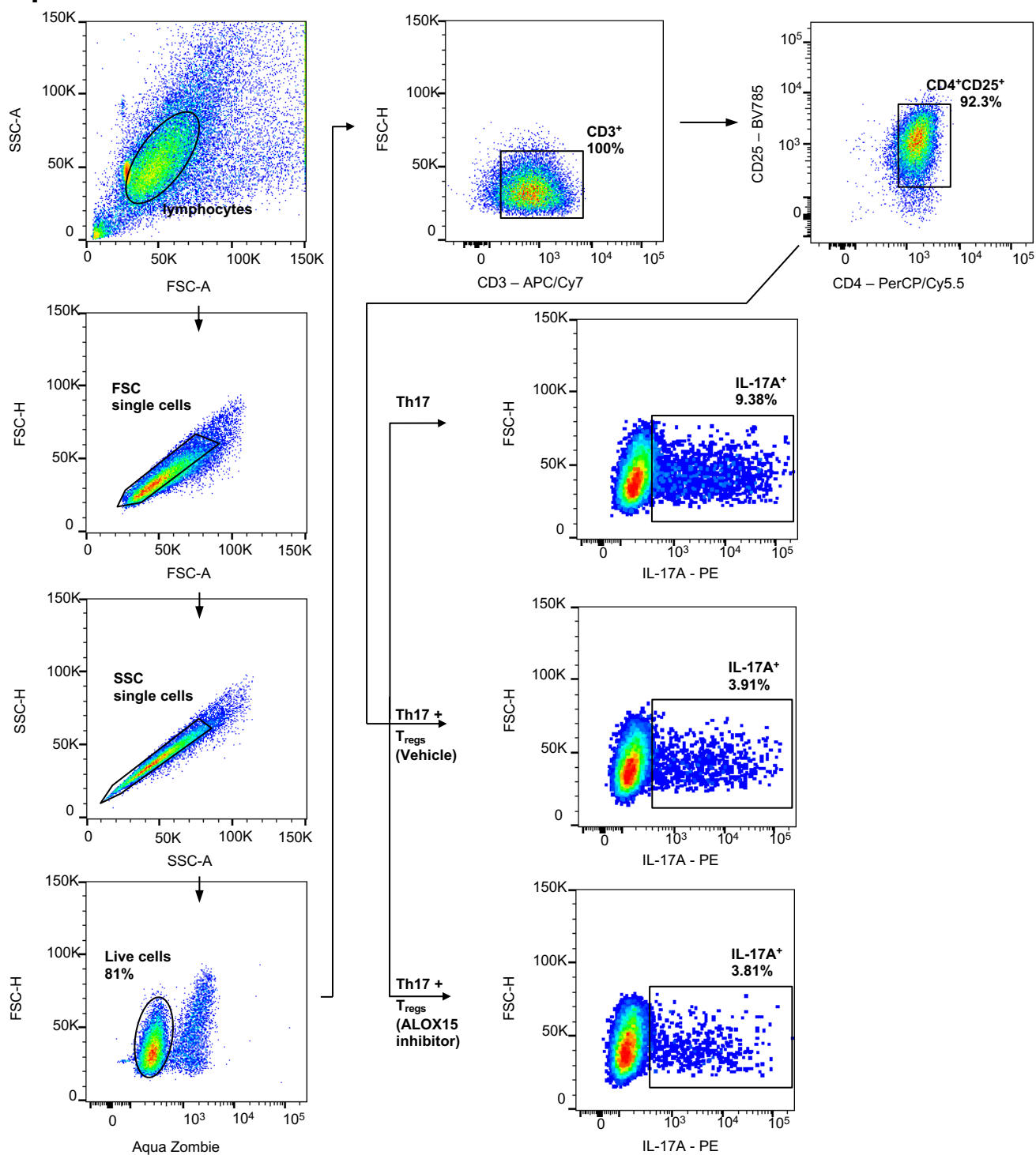

Figure S2

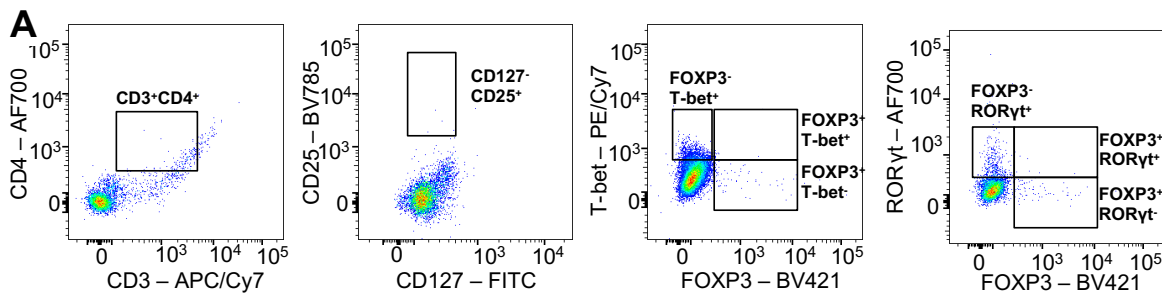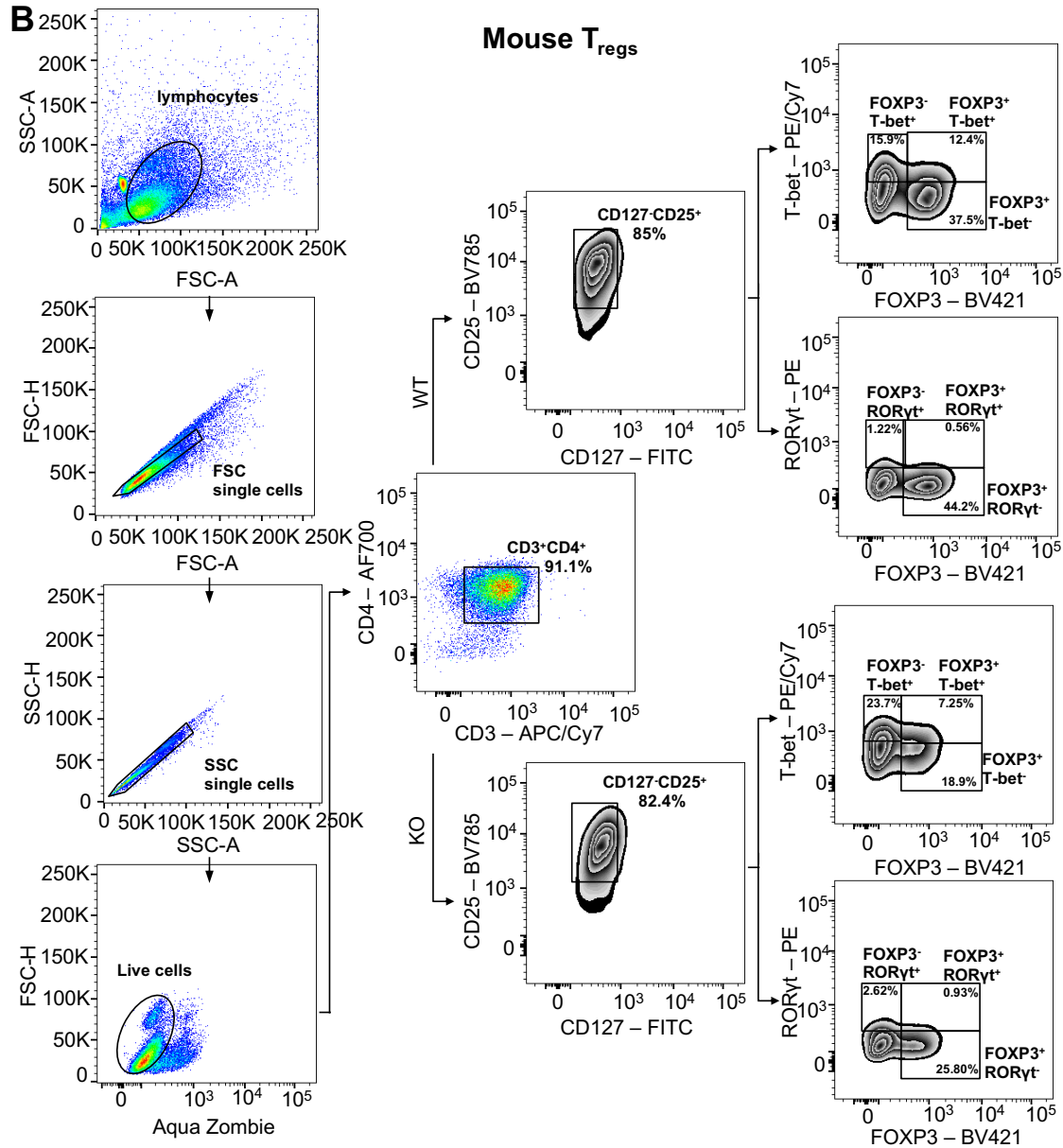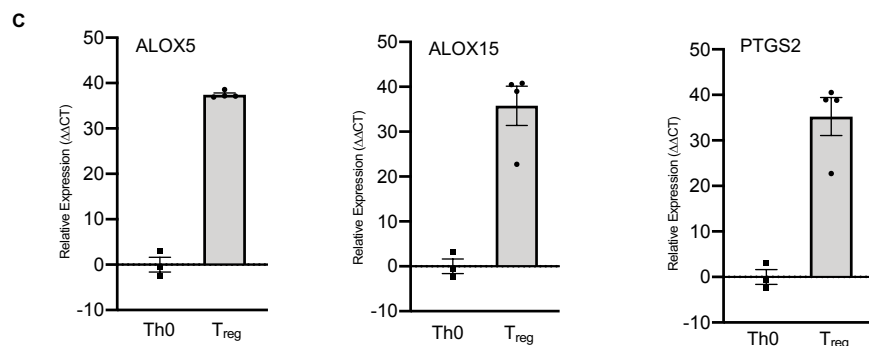

Figure S3

**A**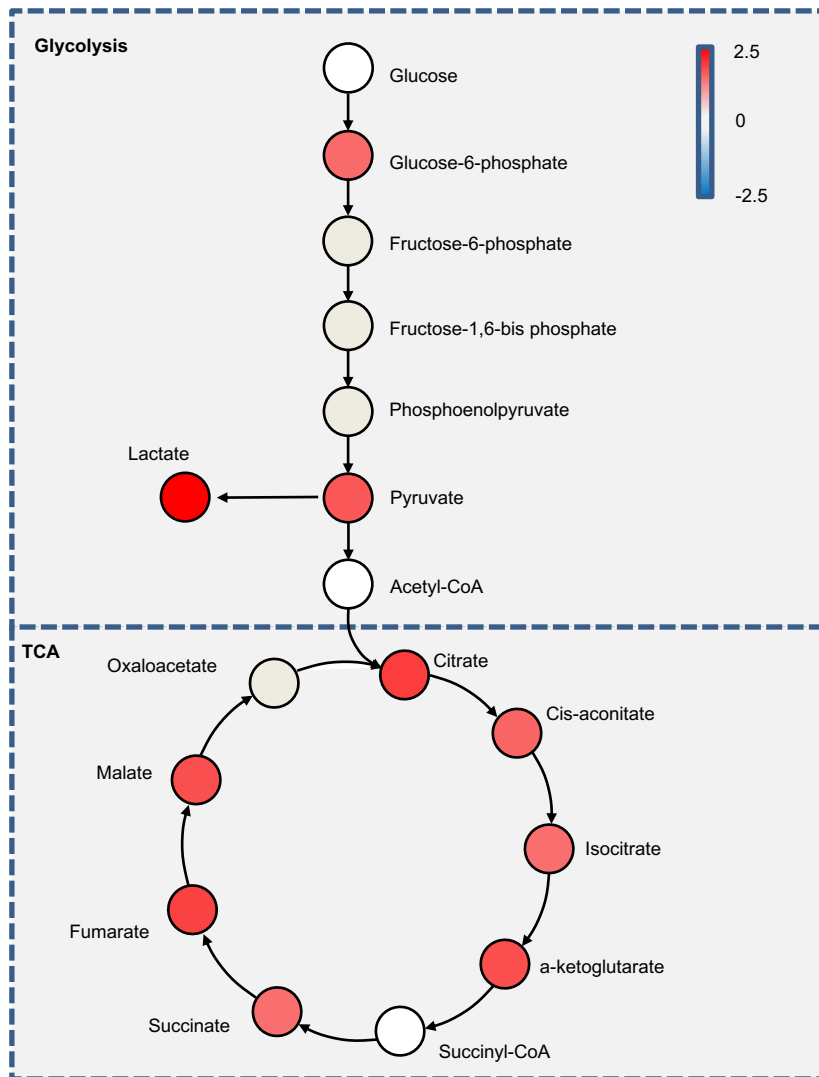**B**

Human

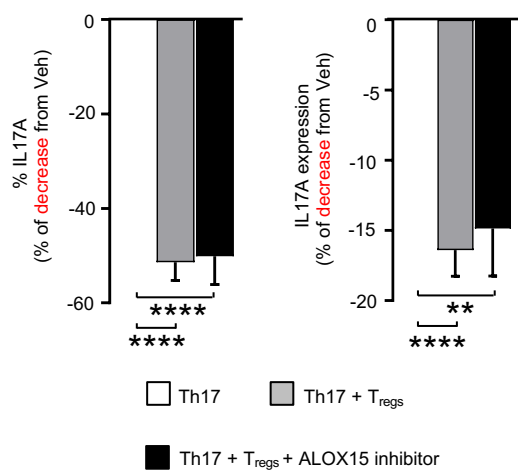**C**

Mouse

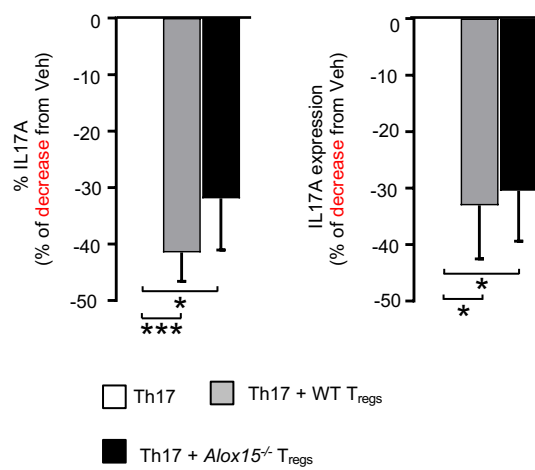

Figure S4

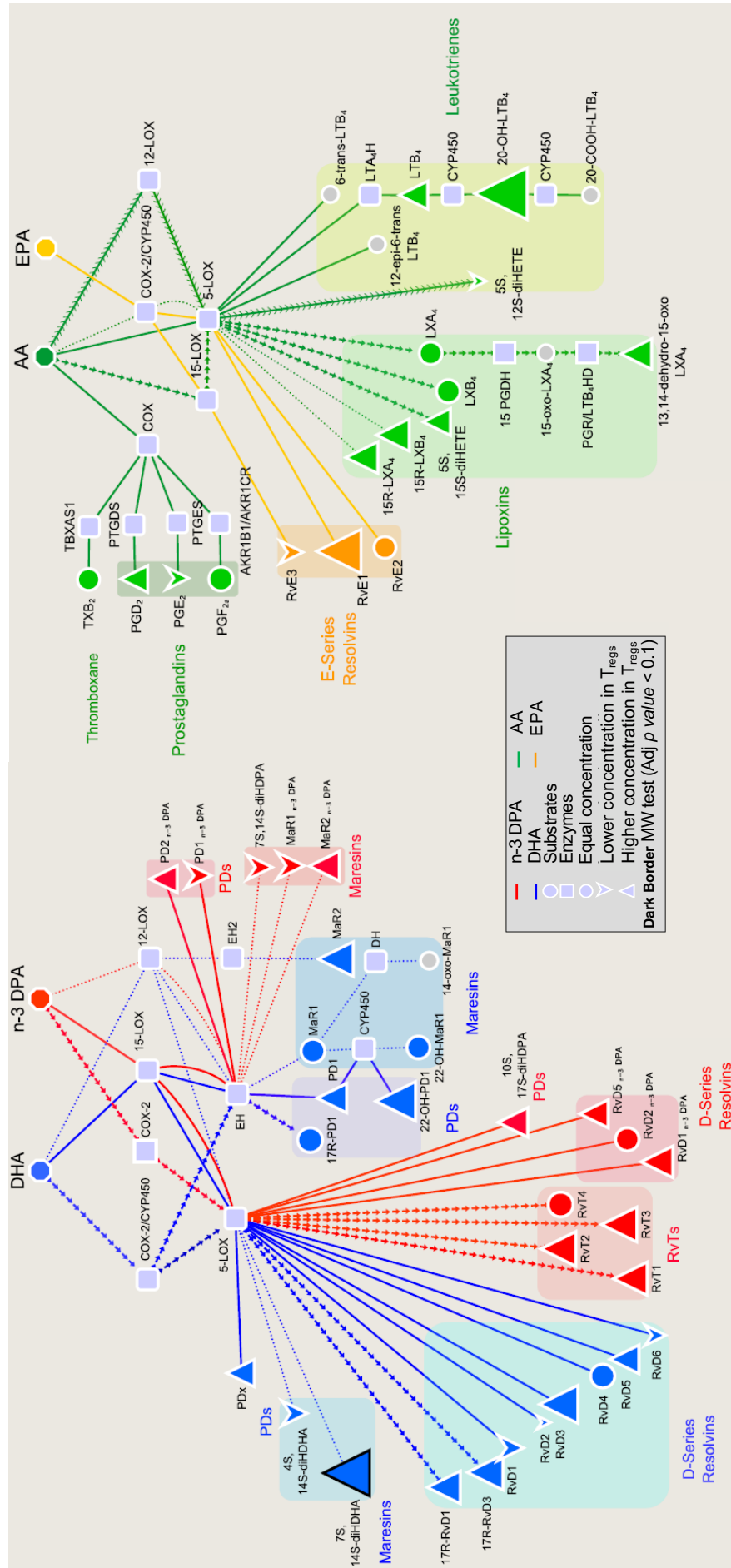

**A**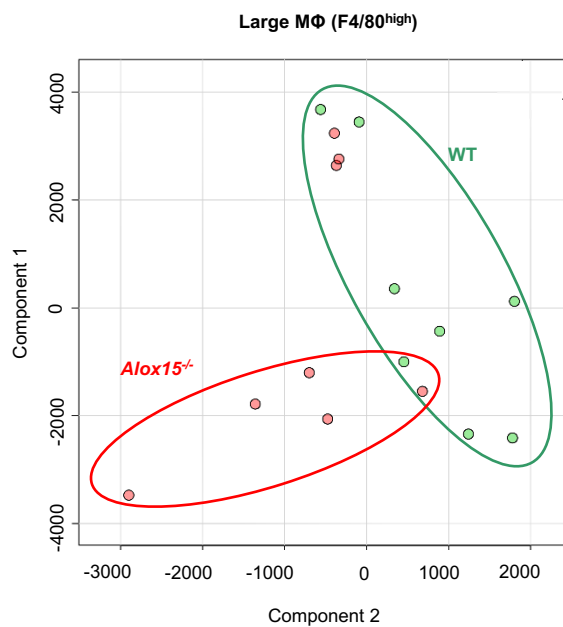**B**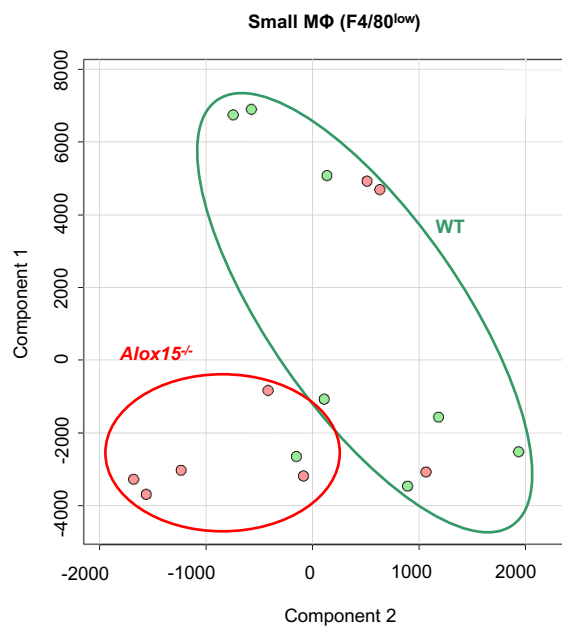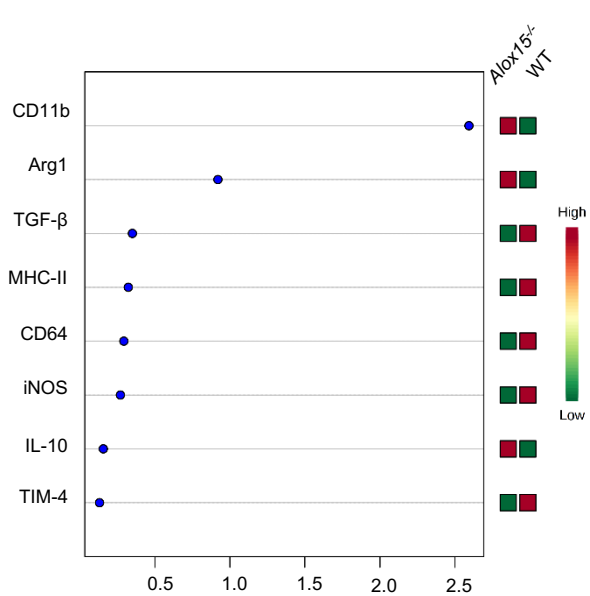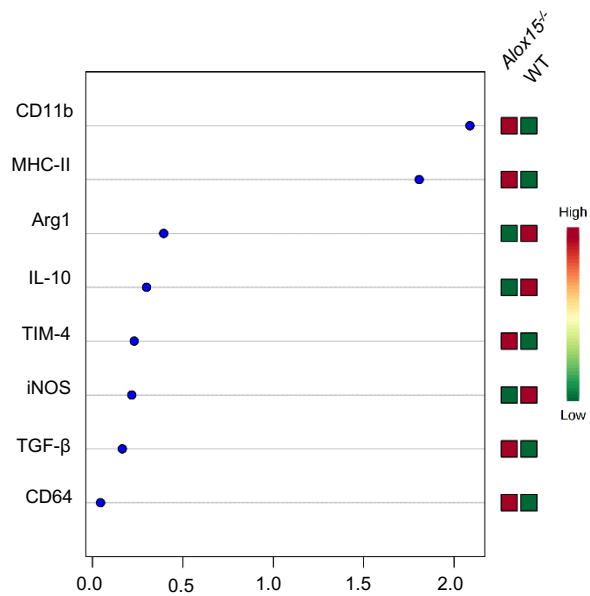

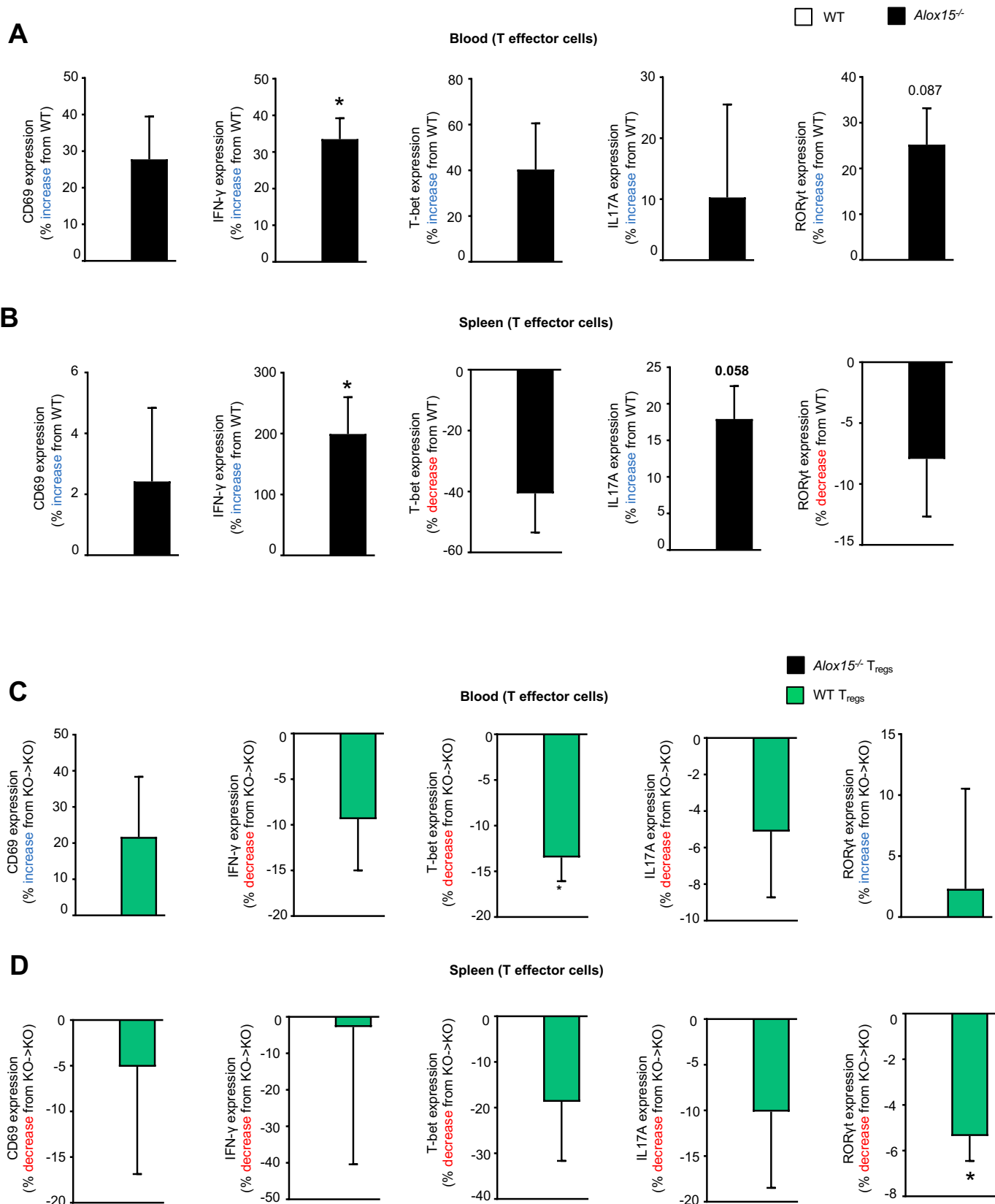

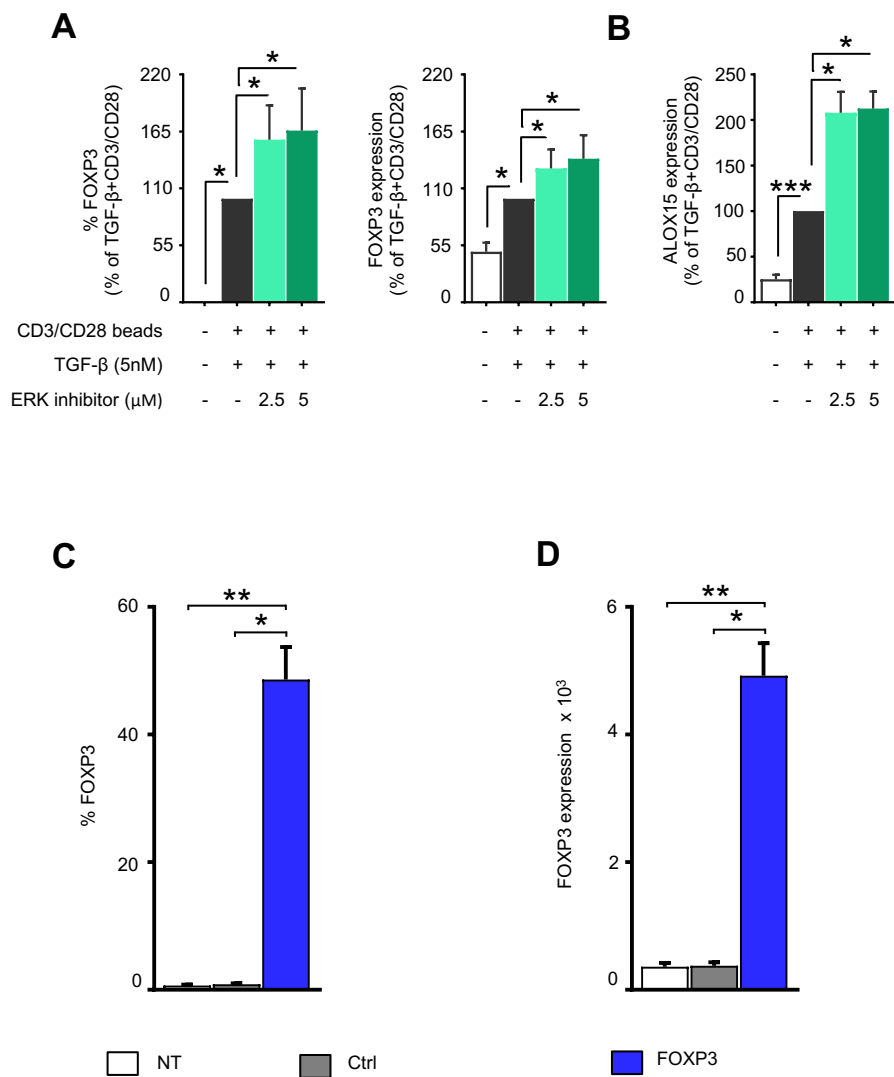

Supplement: Supplementary file 2 — Supplemental Figures [file 41418_2021_807_MOESM2_ESM.pdf]
