# Supplementary material for: Loss of 15-lipoxygenase disrupts Treg differentiation altering their pro-resolving functions
Source: Cell Death Differ. 2021 May 27;28(11):3140–60. doi: 10.1038/s41418-021-00807-x (PMC8563763; doi:10.1038/s41418-021-00807-x)
Supplement: Supplementary file 3 — Supplemental Tables [file 41418_2021_807_MOESM3_ESM.docx]

**Tables:**

**Table S1. Related to Figure 2 – Distinct lipid mediator profiles in Th0 and T_regs_**

|  | **Th0** | | | **T_reg_** | | |
| --- | --- | --- | --- | --- | --- | --- |
| **DHA bioactive metabolome** | ***Mean*** | **±** | ***SEM*** | ***Mean*** | **±** | ***SEM*** |
| RvD1 | 0.25 | ± | 0.18 | 0.06 | ± | 0.07 |
| RvD2 | 0.81 | ± | 0.59 | 0.35 | ± | 0.38 |
| RvD3 |  | - |  | 0.15 | ± | 0.04 |
| RvD4 | 1.36 | ± | 0.81 | 3.72 | ± | 0.83 |
| RvD5 | 0.23 | ± | 0.12 | 0.07 | ± | 0.08 |
| RvD6 | 0.09 | ± | 0.10 |  | - |  |
| 17R-RvD1 | 0.14 | ± | 0.11 | 0.37 | ± | 0.16 |
| 17R -RvD3 |  | - |  | 0.47 | ± | 0.14 |
|  |  |  |  |  |  |  |
| PD1 | 0.20 | ± | 0.11 | 0.26 | ± | 0.17 |
| PDx | 0.04 | ± | 0.03 | 0.90 | ± | 0.80 |
| 17R-PD1 |  |  |  | 0.11 | ± | 0.09 |
| 22-OH-PD1 | 0.04 | ± | 0.04 | 0.41 | ± | 0.28 |
|  |  |  |  |  |  |  |
| MaR1 | 1.82 | ± | 0.43 | 3.62 | ± | 1.41 |
| 7S,14S-diHDHA | 0.13 | ± | 0.14 | 0.86 | ± | 0.36 |
| MaR2 |  | - |  | 1.53 | ± | 1.06 |
| 22-OH-MaR1 |  | - |  |  | - |  |
| 14-oxo-MaR1 | 0.20 | ± | 0.15 | 0.08 | ± | 0.09 |
| **n-3 DPA bioactive metabolome** |  |  |  |  |  |  |
| RvT1 | 0.11 | ± | 0.10 | 0.98 | ± | 0.37 |
| RvT2 |  | - |  | 0.41 | ± | 0.20 |
| RvT3 | 0.04 | ± | 0.04 | 0.16 | ± | 0.13 |
| RvT4 | 0.37 | ± | 0.21 | 0.40 | ± | 0.20 |
|  |  |  |  |  |  |  |
| RvD1_n-3 DPA_ | 0.02 | ± | 0.02 | 0.02 | ± | 0.02 |
| RvD2_n-3 DPA_ | 0.08 | ± | 0.06 | 0.75 | ± | 0.28 |
| RvD5_n-3DPA_ | 0.16 | ± | 0.07 | 0.42 | ± | 0.22 |
|  |  |  |  |  |  |  |
| PD1_n-3 DPA_ | 0.20 | ± | 0.08 | 0.26 | ± | 0.12 |
| PD2_n-3 DPA_ | 0.12 | ± | 0.06 | 0.38 | ± | 0.19 |
| 10S, 17S-diHDPA | 0.12 | ± | 0.06 | 0.24 | ± | 0.14 |
|  |  |  |  |  |  |  |
| MaR1_n-3 DPA_ |  | - |  | 0.07 | ± | 0.07 |
| MaR2_n-3 DPA_ | 0.48 | ± | 0.38 | 8.72 | ± | 3.22 |
| 7S, 14S-diHDPA | 0.69 | ± | 0.17 | 0.94 | ± | 0.36 |
| **EPA bioactive metabolome** |  |  |  |  |  |  |
| RvE1 |  | - |  | 1.36 | ± | 1.05 |
| RvE2 | 0.08 | ± | 0.07 | 2.76 | ± | 0.87 |
| RvE3 | 0.99 | ± | 0.70 | 1.17 | ± | 0.89 |
| **AA bioactive metabolome** |  |  |  |  |  |  |
| LXA_4_ | 0.46 | ± | 0.36 | 1.98 | ± | 2.17 |
| LXB_4_ | 2.63 | ± | 1.85 | 2.24 | ± | 2.45 |
| 5S,15S-diHETE | 9.30 | ± | 3.33 | 3.89 | ± | 4.26 |
| 15-epi-LXA_4_ | 0.23 | ± | 0.19 | 0.30 | ± | 0.21 |
| 15-epi-LXB_4_ | 0.48 | ± | 0.17 | 2.18 | ± | 0.80 |
| 13,14-dehydro-15-oxo-LXA_4_ | 9.65 | ± | 3.85 | 21.93 | ± | 8.07 |
|  |  |  |  |  |  |  |
| LTB_4_ | 0.20 | ± | 0.22 | 0.10 | ± | 0.06 |
| 5S,12S-diHETE | 0.08 | ± | 0.08 |  | - |  |
| 20-OH-LTB_4_ |  | - |  | 2.01 | ± | 0.77 |
|  |  |  |  |  |  |  |
| PGD_2_ | 0.28 | ± | 0.09 | 0.98 | ± | 0.46 |
| PGE_2_ | 0.04 | ± | 0.04 | 0.50 | ± | 0.50 |
| PGF_2a_ | 0.22 | ± | 0.09 | 0.83 | ± | 0.14 |
| TXB_2_ | 0.76 | ± | 0.35 | 0.64 | ± | 0.18 |

Naive CD4^+^ T lymphocytes (Th0) were isolated from healthy volunteers and either placed in ice-cold methanol containing deuterium labelled internal standards or differentiated to T_regs_ (see methods for details). At the end of the incubations, cells were quenched with ice-cold methanol containing deuterium labelled internal standards and lipid mediators were identified and quantified using lipid mediator profiling. Results represent concentrations of mediators found in T-cell incubations less the amount already present in the culture media. Results mean ± s.e.m. of n=6 donors from three distinct experiments. Concentrations were calculated subtracting concentrations found in culture media without cells and presented as pg/1.5 x 10^6^ cells. – denotes that concentrations in the T-cell incubations were equal or below to those found in media

**Table S2: Inhibition of ALOX15 during T_reg_ differentiation reduced SPM formation.**

|  | **T_regs_ + Vehicle** | | | **T_regs_ + ALOX15 Inh** | | |
| --- | --- | --- | --- | --- | --- | --- |
| **DHA bioactive metabolome** | ***Mean*** | **±** | ***SEM*** | ***Mean*** | **±** | ***SEM*** |
| RvD1 | 3.30 | ± | 1.25 | 1.41 | ± | 0.78 |
| RvD2 | 1.30 | ± | 0.80 |  | - |  |
| RvD3 | 0.40 | ± | 0.08 | 0.09 | ± | 0.04 |
| RvD4 |  | - |  |  | - |  |
| RvD5 | 0.37 | ± | 0.08 | 0.24 | ± | 0.07 |
| RvD6 | 0.11 | ± | 0.05 | 0.03 | ± | 0.02 |
| 17R-RvD1 | 0.42 | ± | 0.17 | 0.05 | ± | 0.02 |
| 17R -RvD3 | 0.06 | ± | 0.05 | 0.05 | ± | 0.03 |
|  |  |  |  |  |  |  |
| PD1 |  | - |  |  | - |  |
| PDx | 0.07 | ± | 0.03 | 0.06 | ± | 0.05 |
| 17R-PD1 | 0.05 | ± | 0.03 | 0.02 | ± | 0.01 |
| 22-OH-PD1 | 0.01 | ± | 0.01 |  | - |  |
|  |  |  |  |  |  |  |
| MaR1 | 1.08 | ± | 0.92 |  | - |  |
| 7S,14S-diHDHA | 1.83 | ± | 0.47 | 1.72 | ± | 0.44 |
| MaR2 | 0.02 | ± | 0.02 | 0.03 | ± | 0.03 |
| 22-OH-MaR1 |  | - |  |  | - |  |
| 4S, 14S-diHDHA |  | - |  | 0.50 | ± | 0.26 |
| **n-3 DPA bioactive metabolome** |  |  |  |  |  |  |
| RvT1 | 0.18 | ± | 0.10 | 0.12 | ± | 0.07 |
| RvT2 | 0.37 | ± | 0.20 | 0.06 | ± | 0.05 |
| RvT3 | 0.26 | ± | 0.17 | 0.05 | ± | 0.05 |
| RvT4 | 0.28 | ± | 0.15 | 0.03 | ± | 0.02 |
|  |  |  |  |  |  |  |
| RvD1_n-3 DPA_ | 0.85 | ± | 0.27 | 0.31 | ± | 0.17 |
| RvD2_n-3 DPA_ | 2.16 | ± | 1.22 | 0.53 | ± | 0.21 |
| RvD5_n-3DPA_ | 0.57 | ± | 0.18 |  | - |  |
|  |  |  |  |  |  |  |
| PD1_n-3 DPA_ | 0.23 | ± | 0.05 | 0.05 | ± | 0.04 |
| PD2_n-3 DPA_ | 0.13 | ± | 0.07 |  | - |  |
| 10S, 17S-diHDPA | 0.07 | ± | 0.04 |  | - |  |
|  |  |  |  |  |  |  |
| MaR1_n-3 DPA_ | 0.47 | ± | 0.39 |  | - |  |
| MaR2_n-3 DPA_ | 4.73 | ± | 2.10 | 9.13 | ± | 2.15 |
| 7S, 14S-diHDPA | 0.04 | ± | 0.04 |  | - |  |
| **EPA bioactive metabolome** |  |  |  |  |  |  |
| RvE1 | 0.41 | ± | 0.35 |  | - |  |
| RvE2 | 1.13 | ± | 0.59 | 0.77 | ± | 0.30 |
| RvE3 | 0.10 | ± | 0.05 | 0.69 | ± | 0.59 |
| **AA bioactive metabolome** |  |  |  |  |  |  |
| LXA_4_ | 0.75 | ± | 0.29 | 1.53 | ± | 0.53 |
| LXB_4_ | 1.94 | ± | 0.77 |  | - |  |
| 5S,15S-diHETE |  | - |  |  | - |  |
| 15-epi-LXA_4_ |  | - | 0.00 |  | - |  |
| 15-epi-LXB_4_ | 1.36 | ± | 1.15 | 6.29 | ± | 2.99 |
| 13,14-dehydro-15-oxo-LXA_4_ |  | - |  |  | - |  |
|  |  |  |  |  |  |  |
| LTB_4_ | 0.02 | ± | 0.01 | 0.04 | ± | 0.04 |
| 5S,12S-diHETE |  | - |  |  | - |  |
| 20-OH-LTB_4_ | 0.10 | ± | 0.09 |  | - |  |
|  |  |  |  |  |  |  |
| PGD_2_ |  | - |  |  | - |  |
| PGE_2_ |  | - |  |  | - |  |
| PGF_2a_ | 7.99 | ± | 1.59 | 3.83 | ± | 2.11 |
| TXB_2_ | 6.29 | ± | 0.44 | 2.23 | ± | 0.89 |

Naive CD4^+^ T lymphocytes (Th0) were differentiated to T_regs_ (see methods for details) in the presence of an ALOX15 inhibitor (PD146176; 5 μM). At the end of the incubations, cells were quenched with ice-cold methanol containing deuterium labelled internal standards and lipid mediators were identified and quantified using lipid mediator profiling. Concentrations were calculated subtracting concentrations found in culture media without cells and presented as pg/1.5 x 10^6^ cells. – denotes that concentrations in the T-cell incubations were equal or below to those found in media. Results mean ± s.e.m. of n=7 donors from two distinct experiments.

**Table S3: Reduced SPM levels in Tregs differentiated from *Alox15*^-/-^ naive T-cells when compared with those differentiated from WT naïve T-cells.**

|  | **WT T_regs_** | | | ***Alox15^-^*^/-^ T_regs_** | | |
| --- | --- | --- | --- | --- | --- | --- |
| **DHA bioactive metabolome** | ***Mean*** | **±** | ***SEM*** | ***Mean*** | **±** | ***SEM*** |
| RvD1 |  | - |  |  | - |  |
| RvD2 | 3.11 | ± | 0.71 | 0.46 | ± | 0.13 |
| RvD3 | 1.39 | ± | 0.41 | 0.03 | ± | 0.02 |
| RvD4 | 0.22 | ± | 0.17 | 0.22 | ± | 0.14 |
| RvD5 | 0.72 | ± | 0.14 | 0.35 | ± | 0.12 |
| RvD6 | 0.02 | ± | 0.02 |  | - |  |
| 17R-RvD1 | 0.17 | ± | 0.13 | 0.58 | ± | 0.11 |
| 17R -RvD3 | 0.56 | ± | 0.24 |  | - |  |
|  |  |  |  |  |  |  |
| PD1 |  | - |  |  | - |  |
| PDx | 0.11 | ± | 0.06 | 0.03 | ± | 0.02 |
| 17R-PD1 |  | - |  |  | - |  |
| 22-OH-PD1 |  | - |  |  | - |  |
|  |  |  |  |  |  |  |
| MaR1 |  |  |  |  |  |  |
| 7S,14S-diHDHA | 1.48 | ± | 0.75 |  | - |  |
| MaR2 | 0.99 | ± | 0.27 | 1.54 | ± | 0.64 |
| 22-OH-MaR1 |  | - |  |  | - |  |
| 4S, 14S-diHDHA |  | - |  |  | - |  |
| **n-3 DPA bioactive metabolome** |  |  |  |  |  |  |
| RvT1 | 0.12 | ± | 0.05 | 0.19 | ± | 0.07 |
| RvT2 |  | - |  | 0.60 | ± | 0.11 |
| RvT3 | 0.11 | ± | 0.08 |  | - |  |
| RvT4 | 0.24 | ± | 0.12 |  | - |  |
|  |  |  |  |  |  |  |
| RvD1_n-3 DPA_ |  | - |  |  | - |  |
| RvD2_n-3 DPA_ | 1.23 | ± | 0.39 | 0.45 | ± | 0.16 |
| RvD5_n-3DPA_ | 0.19 | ± | 0.03 |  | - |  |
|  |  |  |  |  |  |  |
| PD1_n-3 DPA_ | 0.11 | ± | 0.04 | 0.14 | ± | 0.05 |
| PD2_n-3 DPA_ | 0.20 | ± | 0.10 | 0.24 | ± | 0.14 |
| 10S, 17S-diHDPA | 0.15 | ± | 0.05 | 0.04 | ± | 0.03 |
|  |  |  |  |  |  |  |
| MaR1_n-3 DPA_ |  | - |  |  | - |  |
| MaR2_n-3 DPA_ | 18.80 | ± | 3.69 | 18.40 | ± | 1.57 |
| 7S, 14S-diHDPA |  | - |  |  | - |  |
| **EPA bioactive metabolome** |  |  |  |  |  |  |
| RvE1 | 0.39 | ± | 0.29 | 0.54 | ± | 0.36 |
| RvE2 | 0.55 | ± | 0.27 | 0.33 | ± | 0.22 |
| RvE3 |  | - |  |  | - |  |
| **AA bioactive metabolome** |  |  |  |  |  |  |
| LXA_4_ | 2.33 | ± | 0.34 | 0.78 | ± | 0.27 |
| LXB_4_ | 80.13 | ± | 8.91 | 42.84 | ± | 2.41 |
| 5S,15S-diHETE | 2.05 | ± | 1.55 | 7.22 | ± | 4.31 |
| 15-epi-LXA_4_ |  | - |  |  | - |  |
| 15-epi-LXB_4_ | 0.33 | ± | 0.25 | 1.53 | ± | 1.00 |
| 13,14-dehydro-15-oxo-LXA_4_ |  | - |  |  | - |  |
|  |  |  |  |  |  |  |
| LTB_4_ | 0.32 | ± | 0.15 | 0.31 | ± | 0.09 |
| 5S,12S-diHETE |  | - |  |  | - |  |
| 20-OH-LTB_4_ | 0.10 | ± | 0.07 | 0.09 | ± | 0.06 |
|  |  |  |  |  |  |  |
| PGD_2_ |  | - |  |  | - |  |
| PGE_2_ | 0.98 | ± | 0.74 | 0.85 | ± | 0.56 |
| PGF_2a_ | 4.52 | ± | 1.15 | 4.89 | ± | 1.21 |
| TXB_2_ | 0.75 | ± | 0.36 | 3.14 | ± | 0.34 |

Naive CD4^+^ T lymphocytes (Th0) were isolated from WT and ALOX15 deficient mice and differentiated to T_regs_ (see methods for details). At the end of the incubations, cells were quenched with ice-cold methanol containing deuterium labelled internal standards and lipid mediators were identified and quantified using lipid mediator profiling. Results represent concentrations of mediators found in T-cell incubations less the amount already present in the culture media. Results mean ± s.e.m. of n=3-4 mice per group and presented as pg/1.5 x 10^6^ cells. – denotes that concentrations in the T-cell incubations were equal or below to those found in media

**Table S4. Related to Figure 4 - Differentially expressed genes of *Alox15*^-/-^ T_regs_ compared to WT T_regs_**

| **Genes** | **logFC** | **logCPM** | ***p value*** | **BH correction (adjust *p value*)** |
| --- | --- | --- | --- | --- |
| Gm14094 | 4.769 | 5.133 | 1.065E-13 | 1.352E-09 |
| Gm5292 | 1.997 | 4.758 | 5.502E-11 | 3.493E-07 |
| AC153864.1 | 2.002 | 4.165 | 9.576E-10 | 4.053E-06 |
| Olfr1033 | -2.572 | 3.376 | 2.634E-09 | 8.364E-06 |
| AA465934 | 2.120 | 5.076 | 3.385E-09 | 8.597E-06 |
| S100a6 | 1.621 | 5.158 | 7.046E-09 | 1.491E-05 |
| Gm4070 | 1.716 | 4.332 | 3.833E-08 | 6.158E-05 |
| Galnt11 | -1.416 | 4.283 | 3.879E-08 | 6.158E-05 |
| Xaf1 | 2.229 | 2.728 | 5.341E-08 | 7.536E-05 |
| Gm8420 | 4.554 | 6.771 | 8.129E-08 | 1.032E-04 |
| Gimap4 | 0.868 | 6.356 | 1.693E-07 | 1.955E-04 |
| Gm16702 | 1.181 | 5.186 | 1.914E-07 | 2.026E-04 |
| Tyrobp | 3.031 | 2.684 | 2.206E-07 | 2.155E-04 |
| Tcrg-C4 | 1.905 | 3.904 | 4.324E-07 | 3.922E-04 |
| Galnt10 | -0.861 | 6.113 | 5.179E-07 | 4.385E-04 |
| Trdc | 1.823 | 5.376 | 8.484E-07 | 6.734E-04 |
| Atox1 | 0.770 | 6.707 | 9.505E-07 | 6.895E-04 |
| Gvin1 | 1.519 | 4.283 | 9.774E-07 | 6.895E-04 |
| Ctla2a | 0.884 | 7.401 | 1.202E-06 | 8.031E-04 |
| Ubb | -0.628 | 9.552 | 1.473E-06 | 8.765E-04 |
| Tcrg-C1 | 2.099 | 2.442 | 1.415E-06 | 8.765E-04 |
| Cma1 | 3.974 | 1.513 | 1.518E-06 | 8.765E-04 |
| Irgm2 | 1.063 | 4.859 | 1.841E-06 | 1.016E-03 |
| Gzmb | 2.736 | 2.401 | 2.061E-06 | 1.090E-03 |
| Ptprd | 0.904 | 5.800 | 2.306E-06 | 1.171E-03 |
| Gm8797 | 0.591 | 8.684 | 3.313E-06 | 1.451E-03 |
| Ccl9 | 2.868 | 3.189 | 3.309E-06 | 1.451E-03 |
| Gpr34 | 1.727 | 2.317 | 3.211E-06 | 1.451E-03 |
| Slc7a8 | 2.934 | 1.614 | 3.169E-06 | 1.451E-03 |
| Slfn1 | 0.703 | 5.822 | 3.448E-06 | 1.459E-03 |
| Thg1l | 1.109 | 4.562 | 3.969E-06 | 1.578E-03 |
| Hdc | 2.547 | 2.519 | 3.975E-06 | 1.578E-03 |
| Ide | -0.657 | 6.511 | 5.156E-06 | 1.926E-03 |
| Thrb | -1.780 | 2.317 | 6.148E-06 | 2.231E-03 |
| Fcer1a | 3.674 | 3.076 | 6.918E-06 | 2.440E-03 |
| Ecm1 | 1.008 | 4.825 | 8.845E-06 | 2.956E-03 |
| Spns3 | 2.886 | 1.280 | 8.768E-06 | 2.956E-03 |
| Gm16867 | -1.902 | 2.034 | 9.326E-06 | 3.037E-03 |
| Arhgef12 | -0.891 | 4.605 | 1.083E-05 | 3.440E-03 |
| Itgae | 1.148 | 4.579 | 1.140E-05 | 3.532E-03 |
| Ms4a6b | 0.707 | 7.036 | 1.233E-05 | 3.728E-03 |
| Pfn1 | 0.653 | 9.514 | 1.486E-05 | 3.979E-03 |
| Gm8885 | 0.739 | 5.674 | 1.393E-05 | 3.979E-03 |
| Ifi27 | 0.853 | 4.977 | 1.548E-05 | 3.979E-03 |
| Gmfg | 0.938 | 4.072 | 1.450E-05 | 3.979E-03 |
| Epsti1 | 1.106 | 3.694 | 1.363E-05 | 3.979E-03 |
| Usp2 | 1.474 | 2.708 | 1.567E-05 | 3.979E-03 |
| Alox5ap | 2.382 | 2.533 | 1.483E-05 | 3.979E-03 |
| Ms4a1 | -2.636 | 1.251 | 1.542E-05 | 3.979E-03 |
| Clec4e | 3.551 | 1.654 | 1.735E-05 | 4.320E-03 |
| Lilrb4a | 2.064 | 3.444 | 1.835E-05 | 4.482E-03 |
| Gm8369 | 0.832 | 5.568 | 1.887E-05 | 4.522E-03 |
| Gbp5 | 0.897 | 4.757 | 2.000E-05 | 4.704E-03 |
| Ms4a4c | 1.215 | 2.927 | 2.354E-05 | 5.435E-03 |
| Prg2 | 3.700 | 4.052 | 2.644E-05 | 5.890E-03 |
| Tcrg-C2 | 1.306 | 3.787 | 2.598E-05 | 5.890E-03 |
| Prss34 | 4.237 | 2.835 | 2.794E-05 | 6.117E-03 |
| Lyrm7 | -1.604 | 2.273 | 2.914E-05 | 6.272E-03 |
| Gata2 | 3.244 | 2.648 | 3.089E-05 | 6.538E-03 |
| Il6 | 2.504 | 0.934 | 3.194E-05 | 6.650E-03 |
| Gbp8 | 0.955 | 3.534 | 4.170E-05 | 8.541E-03 |
| Alox5 | 2.607 | 3.057 | 4.302E-05 | 8.672E-03 |
| Ly6c1 | 2.149 | 5.075 | 5.036E-05 | 9.839E-03 |
| Isg15 | 1.587 | 2.304 | 5.012E-05 | 9.839E-03 |
| Tgtp2 | 0.577 | 6.649 | 5.534E-05 | 1.034E-02 |
| Gbp9 | 0.808 | 5.082 | 5.537E-05 | 1.034E-02 |
| Ctsg | 4.422 | 3.728 | 5.473E-05 | 1.034E-02 |
| Rpl15-ps2 | -0.603 | 7.135 | 5.843E-05 | 1.075E-02 |
| Emilin2 | 2.153 | 1.670 | 6.526E-05 | 1.184E-02 |
| Gm10020 | -0.538 | 7.373 | 7.979E-05 | 1.427E-02 |
| Gzmc | 2.458 | 1.472 | 8.114E-05 | 1.431E-02 |
| Fcgr3 | 2.772 | 1.967 | 8.600E-05 | 1.496E-02 |
| Trim16 | 1.975 | 1.678 | 9.050E-05 | 1.553E-02 |
| Pla2g16 | 0.705 | 5.182 | 9.270E-05 | 1.570E-02 |
| Ifitm1 | 3.469 | 5.216 | 9.561E-05 | 1.586E-02 |
| Plac8 | 3.050 | 3.847 | 9.614E-05 | 1.586E-02 |
| Ly6c2 | 2.646 | 5.033 | 9.883E-05 | 1.589E-02 |
| Dpp4 | 0.894 | 3.626 | 9.885E-05 | 1.589E-02 |
| Gm27177 | -2.326 | 2.073 | 1.022E-04 | 1.622E-02 |
| Gpnmb | 3.794 | 2.728 | 1.136E-04 | 1.759E-02 |
| Ifit3 | 1.583 | 2.672 | 1.132E-04 | 1.759E-02 |
| Igha | 1.033 | 3.382 | 1.163E-04 | 1.779E-02 |
| Pced1b | 0.658 | 4.998 | 1.227E-04 | 1.855E-02 |
| Cd63-ps | 4.544 | 3.189 | 1.283E-04 | 1.856E-02 |
| Rhob | 1.120 | 3.168 | 1.284E-04 | 1.856E-02 |
| Irf7 | 1.258 | 2.958 | 1.251E-04 | 1.856E-02 |
| Cd200r3 | 2.604 | 1.460 | 1.286E-04 | 1.856E-02 |
| Scin | 1.164 | 4.213 | 1.333E-04 | 1.901E-02 |
| Selenow | 0.599 | 6.488 | 1.390E-04 | 1.936E-02 |
| Kynu | -1.098 | 4.160 | 1.396E-04 | 1.936E-02 |
| Entpd4 | -0.857 | 3.950 | 1.410E-04 | 1.936E-02 |
| Gm11290 | 0.888 | 3.676 | 1.418E-04 | 1.936E-02 |
| Ms4a2 | 3.824 | 2.535 | 1.512E-04 | 2.042E-02 |
| Epx | 3.291 | 2.955 | 1.591E-04 | 2.105E-02 |
| Mlkl | 1.476 | 2.486 | 1.579E-04 | 2.105E-02 |
| Usp18 | 1.418 | 3.434 | 1.612E-04 | 2.107E-02 |
| Klrd1 | 1.361 | 2.878 | 1.626E-04 | 2.107E-02 |
| 1110032F04Rik | -0.526 | 7.208 | 1.675E-04 | 2.148E-02 |
| Ifi27l2a | 0.556 | 7.505 | 1.693E-04 | 2.150E-02 |
| Ifi209 | 1.122 | 3.076 | 1.732E-04 | 2.178E-02 |
| Mcpt1 | 3.630 | 4.939 | 1.753E-04 | 2.182E-02 |
| Isg20 | 1.112 | 3.274 | 1.777E-04 | 2.191E-02 |
| Cxcl16 | 1.837 | 1.960 | 1.887E-04 | 2.305E-02 |
| Gm21887 | -0.815 | 6.374 | 1.938E-04 | 2.344E-02 |
| Gbp7 | 1.224 | 3.683 | 2.148E-04 | 2.573E-02 |
| Gm12216 | 1.280 | 2.480 | 2.178E-04 | 2.585E-02 |
| H2-Q7 | 0.589 | 7.290 | 2.222E-04 | 2.611E-02 |
| Apol7e | 1.200 | 2.271 | 2.241E-04 | 2.611E-02 |
| Csf2rb2 | 3.083 | 3.973 | 2.297E-04 | 2.652E-02 |
| Ube2l6 | 1.326 | 2.349 | 2.346E-04 | 2.684E-02 |
| Gm43302 | 0.781 | 5.846 | 2.450E-04 | 2.754E-02 |
| Tubd1 | -1.161 | 2.505 | 2.438E-04 | 2.754E-02 |
| Cd63 | 3.897 | 3.917 | 2.530E-04 | 2.818E-02 |
| Hba-a1 | 4.854 | 7.952 | 2.621E-04 | 2.895E-02 |
| Hba-a2 | 5.009 | 7.826 | 2.708E-04 | 2.964E-02 |
| Fcer1g | 2.355 | 3.902 | 2.783E-04 | 3.021E-02 |
| Gm4294 | -0.465 | 7.466 | 2.856E-04 | 3.074E-02 |
| Cpne2 | 0.721 | 4.641 | 2.960E-04 | 3.152E-02 |
| Tlcd2 | 1.088 | 2.343 | 2.978E-04 | 3.152E-02 |
| Fxr2 | -0.534 | 5.529 | 3.008E-04 | 3.157E-02 |
| Slfn8 | 1.026 | 3.079 | 3.053E-04 | 3.178E-02 |
| Pttg1 | 0.689 | 5.041 | 3.135E-04 | 3.237E-02 |
| Ankrd27 | -0.573 | 4.789 | 3.212E-04 | 3.289E-02 |
| Csf2rb | 3.000 | 4.297 | 3.310E-04 | 3.363E-02 |
| Phlda1 | -0.670 | 4.963 | 3.401E-04 | 3.428E-02 |
| Hbb-bt | 4.979 | 6.985 | 3.473E-04 | 3.472E-02 |
| Cst7 | 1.473 | 3.275 | 3.650E-04 | 3.621E-02 |
| Serpinb1a | 3.884 | 3.047 | 3.774E-04 | 3.715E-02 |
| Zswim7 | 0.767 | 3.979 | 3.981E-04 | 3.889E-02 |
| Stk39 | -0.482 | 5.885 | 4.044E-04 | 3.891E-02 |
| Fgl2 | 2.740 | 3.556 | 4.039E-04 | 3.891E-02 |
| Hbb-bs | 4.920 | 9.033 | 4.173E-04 | 3.915E-02 |
| Mcpt8 | 3.309 | 5.338 | 4.192E-04 | 3.915E-02 |
| Cd9 | 0.676 | 5.290 | 4.132E-04 | 3.915E-02 |
| H2-Q6 | 0.740 | 4.412 | 4.156E-04 | 3.915E-02 |
| Rsad1 | -1.183 | 2.438 | 4.279E-04 | 3.966E-02 |
| Gm4951 | 1.397 | 2.589 | 4.405E-04 | 4.054E-02 |
| Lyz2 | 3.485 | 4.054 | 4.533E-04 | 4.112E-02 |
| Adamtsl4 | 1.620 | 0.994 | 4.505E-04 | 4.112E-02 |
| Ccl6 | 2.604 | 2.560 | 4.638E-04 | 4.177E-02 |
| a | -0.600 | 6.732 | 4.940E-04 | 4.418E-02 |
| Itga2b | 1.775 | 1.174 | 5.434E-04 | 4.826E-02 |
| Padi2 | 0.850 | 4.005 | 5.506E-04 | 4.856E-02 |
| Bst2 | 0.557 | 6.168 | 5.653E-04 | 4.951E-02 |

**Table S5. Related to Figure S4 –ALOX15-derived lipid mediators are upregulated 24h into the T_reg_ differentiation program**

|  | **Th0** | | | **T_regs_ (24h after**  **start of differentiation)** | | |
| --- | --- | --- | --- | --- | --- | --- |
| **DHA bioactive metabolome** | ***Mean*** | **±** | ***SEM*** | ***Mean*** | **±** | ***SEM*** |
| RvD1 | 0.19 | ± | 0.14 | 0.08 | ± | 0.06 |
| RvD2 | 1.09 | ± | 0.61 |  | - |  |
| RvD3 | 0.03 | ± | 0.03 | 2.30 | ± | 1.13 |
| RvD4 | 1.02 | ± | 0.63 | 1.47 | ± | 0.63 |
| RvD5 | 0.18 | ± | 0.10 | 0.64 | ± | 0.26 |
| RvD6 | 0.07 | ± | 0.07 |  |  |  |
| 17R-RvD1 | 0.10 | ± | 0.08 | 1.35 | ± | 1.05 |
| 17R -RvD3 | 0.07 | ± | 0.07 | 2.26 | ± | 2.18 |
|  |  |  |  |  |  |  |
| PD1 | 0.15 | ± | 0.09 | 0.67 | ± | 0.45 |
| PDx | 0.16 | ± | 0.14 | 0.40 | ± | 0.27 |
| 17R-PD1 |  | - |  | 0.53 | ± | 0.53 |
| 22-OH-PD1 | 0.03 | ± | 0.03 | 0.05 | ± | 0.06 |
|  |  |  |  |  |  |  |
| MaR1 | 1.37 | ± | 0.44 | 1.77 | ± | 1.53 |
| 7S,14S-diHDHA | 0.09 | ± | 0.10 | 2.38 | ± | 1.02 |
| MaR2 |  | - |  | 3.85 | ± | 1.01 |
| 22-OH-MaR1 |  | - |  |  | - |  |
| 4S, 14S-diHDHA | 0.15 | ± | 0.11 | 0.05 | ± | 0.06 |
| **n-3 DPA bioactive metabolome** |  |  |  |  |  |  |
| RvT1 | 0.09 | ± | 0.07 | 1.37 | ± | 1.32 |
| RvT2 |  | - |  | 0.12 | ± | 0.13 |
| RvT3 | 0.03 | ± | 0.03 | 0.80 | ± | 0.86 |
| RvT4 | 0.28 | ± | 0.17 | 0.54 | ± | 0.21 |
|  |  |  |  |  |  |  |
| RvD1_n-3 DPA_ | 0.09 | ± | 0.08 | 0.88 | ± | 0.82 |
| RvD2_n-3 DPA_ | 0.06 | ± | 0.04 | 0.07 | ± | 0.04 |
| RvD5_n-3DPA_ | 0.16 | ± | 0.09 | 0.50 | ± | 0.14 |
|  |  |  |  |  |  |  |
| PD1_n-3 DPA_ | 0.36 | ± | 0.12 | 0.16 | ± | 0.07 |
| PD2_n-3 DPA_ | 0.79 | ± | 0.47 | 1.78 | ± | 1.23 |
| 10S, 17S-diHDPA | 0.09 | ± | 0.05 | 0.48 | ± | 0.35 |
|  |  |  |  |  |  |  |
| MaR1_n-3 DPA_ | 0.37 | ± | 0.39 | 0.27 | ± | 0.19 |
| MaR2_n-3 DPA_ | 1.75 | ± | 1.46 | 8.53 | ± | 2.77 |
| 7S, 14S-diHDPA | 1.10 | ± | 0.57 | 0.39 | ± | 0.24 |
| **EPA bioactive metabolome** |  |  |  |  |  |  |
| RvE1 |  | - |  | 0.52 | ± | 0.56 |
| RvE2 | 1.66 | ± | 1.14 | 1.71 | ± | 0.81 |
| RvE3 | 0.74 | ± | 0.53 | 0.33 | ± | 0.35 |
| **AA bioactive metabolome** |  |  |  |  |  |  |
| LXA_4_ | 0.34 | ± | 0.27 | 0.43 | ± | 0.34 |
| LXB_4_ | 3.27 | ± | 1.59 | 4.68 | ± | 2.05 |
| 5S,15S-diHETE | 8.92 | ± | 2.86 | 19.63 | ± | 7.65 |
| 15-epi-LXA_4_ | 1.02 | ± | 0.67 | 7.03 | ± | 4.93 |
| 15-epi-LXB_4_ | 19.55 | ± | 12.43 | 153.21 | ± | 91.72 |
| 13,14-dehydro-15-oxo-LXA_4_ | 0.43 | ± | 0.29 | 2.12 | ± | 2.04 |
|  |  |  |  |  |  |  |
| LTB_4_ | 0.15 | ± | 0.16 | 0.46 | ± | 0.15 |
| 5S,12S-diHETE | 0.06 | ± | 0.06 |  | - |  |
| 20-OH-LTB_4_ |  | - |  | 3.27 | ± | 1.38 |
|  |  |  |  |  |  |  |
| PGD_2_ | 0.30 | ± | 0.09 | 0.11 | ± | 0.06 |
| PGE_2_ | 0.22 | ± | 0.20 | 0.46 | ± | 0.26 |
| PGF_2a_ | 0.32 | ± | 0.10 | 0.54 | ± | 0.13 |
| TXB_2_ | 3.33 | ± | 1.82 | 3.54 | ± | 1.33 |

Naive CD4^+^ T lymphocytes (Th0) were isolated from healthy volunteers and either placed in ice-cold methanol containing deuterium labelled internal standards or incubated with T_reg_ cocktail for the indicated time intervals. At the end of the incubations, cells were quenched with ice-cold methanol containing deuterium labelled internal standards and lipid mediators were identified and quantified using lipid mediator profiling. Results represent concentrations of mediators found in T-cell incubations less the amount already present in the culture media. Results mean ± s.e.m. of n=4 donors per group from two distinct experiments and presented as pg/1.5 x 10^6^ cells. – denotes that concentrations in the T-cell incubations were equal or below to those found in media.

**Table S6. Related to Figure 7 – Rapid upregulation of ALOX15-derived lipid mediators during T_reg_ differentiation**

|  | **Th0** | | | **T_regs_ (1h after**  **start of differentiation)** | | |
| --- | --- | --- | --- | --- | --- | --- |
| **DHA bioactive metabolome** | ***Mean*** | **±** | ***SEM*** | ***Mean*** | **±** | ***SEM*** |
| RvD1 | 0.19 | ± | 0.14 | 0.97 | ± | 0.68 |
| RvD2 | 1.09 | ± | 0.61 | 0.70 | ± | 0.35 |
| RvD3 | 0.01 | ± | 0.01 | 1.36 | ± | 0.81 |
| RvD4 | 1.02 | ± | 0.63 | 1.69 | ± | 0.80 |
| RvD5 | 0.18 | ± | 0.10 | 0.48 | ± | 0.21 |
| RvD6 | 0.07 | ± | 0.07 | 0.11 | ± | 0.07 |
| 17R-RvD1 | 0.10 | ± | 0.08 | 1.75 | ± | 1.27 |
| 17R -RvD3 | 0.07 | ± | 0.07 | 2.00 | ± | 1.51 |
|  |  |  |  |  |  |  |
| PD1 | 0.15 | ± | 0.09 | 0.14 | ± | 0.06 |
| PDx | 0.16 | ± | 0.14 | 0.93 | ± | 0.27 |
| 17R-PD1 |  |  |  | 0.06 | ± | 0.04 |
| 22-OH-PD1 | 0.03 | ± | 0.03 | 0.12 | ± | 0.09 |
|  |  |  |  |  |  |  |
| MaR1 | 6.50 | ± | 5.29 | 2.32 | ± | 0.96 |
| 7S,14S-diHDHA | 0.09 | ± | 0.10 | 1.08 | ± | 0.68 |
| MaR2 |  | - |  | 4.45 | ± | 1.44 |
| 22-OH-MaR1 |  | - |  |  | - |  |
| 4S, 14S-diHDHA | 0.15 | ± | 0.11 | 0.11 | ± | 0.08 |
| **n-3 DPA bioactive metabolome** |  |  |  |  |  |  |
| RvT1 | 0.09 | ± | 0.07 | 2.01 | ± | 1.75 |
| RvT2 |  | - |  | 0.22 | ± | 0.19 |
| RvT3 | 0.03 | ± | 0.03 | 0.15 | ± | 0.12 |
| RvT4 | 0.28 | ± | 0.17 | 1.47 | ± | 0.50 |
|  |  |  |  |  |  |  |
| RvD1_n-3 DPA_ | 0.09 | ± | 0.08 | 0.01 | ± | 0.01 |
| RvD2_n-3 DPA_ | 0.06 | ± | 0.04 | 2.35 | ± | 2.34 |
| RvD5_n-3DPA_ | 0.21 | ± | 0.09 | 0.80 | ± | 0.21 |
|  |  |  |  |  |  |  |
| PD1_n-3 DPA_ | 0.36 | ± | 0.12 | 0.04 | ± | 0.03 |
| PD2_n-3 DPA_ | 0.79 | ± | 0.47 | 0.36 | ± | 0.11 |
| 10S, 17S-diHDPA | 0.09 | ± | 0.05 | 0.35 | ± | 0.13 |
|  |  |  |  |  |  |  |
| MaR1_n-3 DPA_ | 0.37 | ± | 0.39 | 0.20 | ± | 0.11 |
| MaR2_n-3 DPA_ | 1.75 | ± | 1.46 | 3.67 | ± | 1.27 |
| 7S, 14S-diHDPA | 1.10 | ± | 0.57 | 0.54 | ± | 0.15 |
| **EPA bioactive metabolome** |  |  |  |  |  |  |
| RvE1 |  | - |  | 1.07 | ± | 1.14 |
| RvE2 | 1.66 | ± | 1.14 | 0.98 | ± | 0.36 |
| RvE3 | 0.74 | ± | 0.53 | 0.39 | ± | 0.29 |
| **AA bioactive metabolome** |  |  |  |  |  |  |
| LXA_4_ | 0.34 | ± | 0.27 | 2.81 | ± | 1.36 |
| LXB_4_ | 14.96 | ± | 11.63 | 36.47 | ± | 32.03 |
| 5S,15S-diHETE | 8.92 | ± | 2.86 | 4.00 | ± | 1.67 |
| 15-epi-LXA_4_ | 12.67 | ± | 13.10 | 55.42 | ± | 49.34 |
| 15-epi-LXB_4_ | 7.50 | ± | 3.14 | 11.13 | ± | 4.34 |
| 13,14-dehydro-15-oxo-LXA_4_ | 0.83 | ± | 0.70 | 5.17 | ± | 3.33 |
|  |  |  |  |  |  |  |
| LTB_4_ | 0.15 | ± | 0.16 | 0.65 | ± | 0.27 |
| 5S,12S-diHETE | 0.06 | ± | 0.06 | 0.16 | ± | 0.12 |
| 20-OH-LTB_4_ |  | - |  |  | - |  |
|  |  |  |  |  |  |  |
| PGD_2_ | 0.30 | ± | 0.09 | 0.26 | ± | 0.11 |
| PGE_2_ | 0.22 | ± | 0.20 | 0.52 | ± | 0.19 |
| PGF_2a_ | 0.32 | ± | 0.10 | 0.70 | ± | 0.26 |
| TXB_2_ | 3.33 | ± | 1.82 | 1.71 | ± | 1.15 |

Naive CD4^+^ T lymphocytes (Th0) were isolated from healthy volunteers and either incubated with T_reg_ cocktail or PBS for 1h (37^o^C). At the end of the incubations, cells were quenched with ice-cold methanol containing deuterium labelled internal standards and lipid mediators were identified and quantified using lipid mediator profiling. Results represent concentrations of mediators found in T-cell incubations less the amount already present in the culture media. Results mean ± s.e.m. of n=6 donors from three distinct experiments. – denotes that concentrations in the T-cell incubations were equal or below to those found in media.

**Table S7. Related to Methods section: List of MRM transitions employed for identification of glycolytic. TCA intermediates and internal standards**

| **Compound** | **Q1** | **Q3** |
| --- | --- | --- |
| ^13^C_6_-Glucose | 185.2 | 92.1 |
| Glucose | 179.1 | 89.1 |
| Fructose | 179.1 | 89.1 |
| Glucose-6P | 259.2 | 97.1 |
| Fuctose-6P | 259.2 | 97.1 |
| Fructose1.6bisP | 339.1 | 97.1 |
| DiOHacetone-P | 169.1 | 97.1 |
| Glyceraldehyde-3P | 169.1 | 97.1 |
| 3P Glycerate | 185.1 | 79.1 |
| PenolPyruvate | 167.1 | 79.1 |
| ^13^C_2_-Citrate | 193.2 | 112.1 |
| ^13^C_2_-Fumarate | 117.2 | 73.1 |
| a-keto glutarate | 145.0 | 101.1 |
| Citrate | 191.0 | 111.1 |
| Iso-citrate | 191.0 | 111.1 |
| Succinate | 117.2 | 73.3 |
| Malate | 133.1 | 115.4 |
| Cis-aconitate | 173.1 | 85.1 |
| Pyruvate | 87.1 | 87.1 |
| Oxaloacetate | 131.1 | 87.0 |
| Fumarate | 115.1 | 71.1 |
| Lactate | 89.2 | 43.0 |
